# Supplementary material for: CODIFI (Concordance in Diabetic Foot Ulcer Infection): a cross-sectional study of wound swab versus tissue sampling in infected diabetic foot ulcers in England
Source: BMJ Open. 2018 Jan 31;8(1):e019437. doi: 10.1136/bmjopen-2017-019437 (PMC5879729; doi:10.1136/bmjopen-2017-019437)
Supplement: Author's manuscript [file bmjopen-2017-019437.draft_revisions.pdf]

BMJ Open is committed to open peer review. As part of this commitment we make the peer review history of every article we publish publicly available.

When an article is published we post the peer reviewers' comments and the authors' responses online. We also post the versions of the paper that were used during peer review. These are the versions that the peer review comments apply to.

The versions of the paper that follow are the versions that were submitted during the peer review process. They are not the versions of record or the final published versions. They should not be cited or distributed as the published version of this manuscript.

BMJ Open is an open access journal and the full, final, typeset and author-corrected version of record of the manuscript is available on our site with no access controls, subscription charges or pay-per-view fees (http://bmjopen.bmj.com).

If you have any questions on BMJ Open's open peer review process please email editorial.bmjopen@bmj.com

BMJ Open

The CODIFI study of swab versus tissue sampling in infected diabetic foot ulcers - (CODIFI: Concordance In Diabetic Foot Ulcer Infection)

Journal:	*BMJ Open*
Manuscript ID	bmjopen-2017-019437
Article Type:	Research
Date Submitted by the Author:	31-Aug-2017
Complete List of Authors:	Nelson, E. Andrea; University of Leeds, School of Healthcare<br>Wright-Hughes, Alexandra; University of Leeds, Clinical Trials Research Unit<br>Backhouse, Michael; University of Leeds, School of Healthcare<br>Lipsky, BA; Washington University<br>Nixon, Jane; University of Leeds, Clinical Trials Research Unit<br>Bhogal, Moninder; University of Leeds, Clinical Trials Research Unit<br>Reynolds, Catherine; University of Leeds, Clinical Trials Research Unit<br>Brown, Sarah; University of Leeds, Clinical Trials Research Unit
**Primary Subject Heading**:	Diabetes and endocrinology
Secondary Subject Heading:	Research methods
Keywords:	diabetic foot ulcers, swab sampling, tissue sampling, agreement, diabetic foot infection

SCHOLARONE™
Manuscripts

The CODIFI study of swab versus tissue sampling in infected diabetic foot ulcers - (CODIFI: Concordance In Diabetic Foot Ulcer Infection)

E. Andrea Nelson[1] E.A.Nelson@leeds.ac.uk

Alexandra Wright-Hughes[2] A.Wright-Hughes@leeds.ac.uk

Michael R Backhouse[1] M.R.Backhouse@leeds.ac.uk

Benjamin A. Lipsky[3] balipsky@uw.edu

Jane Nixon[2] J.E.Nixon@leeds.ac.uk

Moninder S Bhogal[2] M.S.Bhogal@leeds.ac.uk

Catherine Reynolds[2] C.Reynolds@leeds.ac.uk

Sarah Brown[2] Medsbro@leeds.ac.uk

On behalf of the CODIFI collaborators

The CODIFI collaborators are EA Nelson, J Nixon, S Brown, J Gray, J Firth, C Dowson, E Jude, T Dickie, C Amery, G Sykes, P Vowden, M Edmonds.

Affiliations

[1] School of Healthcare, University of Leeds,

[2] Clinical Trials Research Unit, Leeds Institute of Clinical Trials Research, University of Leeds,

[3] Division of Medical Sciences, University of Oxford

Corresponding author:

E. Andrea Nelson, School of Healthcare

University of Leeds, LS2 9JT

E.A.Nelson@leeds.ac.uk

For peer review only

Abstract

Objective: To determine the extent of agreement and patterns of disagreement between swab and tissue samples in patients with a diabetic foot ulcer (DFU).

Design: Multi-centre, prospective, cross-sectional study.

Setting:Primary and secondary care foot ulcer/diabetic clinics and hospital wards across England.

Participants:Inclusion criteria: Consenting patients aged ≥18 years; diabetes mellitus; suspected infected DFU

Exclusion criteria:clinically inappropriate to take tissue/swab sample.

Interventions:swab culture obtained using Levine's technique; tissue samples collected using a sterile dermal curette or scalpel.

Outcome measures:
Co-primary: reported presence and number of pathogens per sample, prevalence among likely pathogens of resistance to antimicrobials
Secondary: whether a change in therapy was required (blind clinical review panel); adverse events and costs of sampling.

Results: 400 consenting patients (79% female) recruited across 25 centres.
Most prevalent reported pathogens were Staphylococcus *aureus* (43.8%), *Streptococcus* (16.7%), and other aerobic gram-positive cocci (70.6%). At least one potential pathogen was reported from 70.1% of swab and 86.1% of tissue samples. Pathogen results differed between sampling method in 58% of patients, with more pathogens and fewer contaminants reported from tissues.

There was symmetrical disagreement in *S.aureus* and *Pseudomonas;* for other pathogens, all were reported significantly more frequently in tissue than swab samples (p<0.01). Blinded clinicians more often recommended a change in antibiotics based on tissue compared with swab results (increase of 8.9%, 95% CI:2.65,15.3%). Ulcer pain and bleeding occurred more often after tissue sampling than swabbing (pain:9.3%,1.3%; bleeding:6.8%,1.5% for tissue and swab sampling respectively).

Conclusion: Tissue samples more frequently identified most pathogens, and less frequently reported contaminants/colonisers compared with swab samples. Blinded clinicians more often recommended changes in antibiotherapy based on tissue compared with swab

For peer review only

specimens. Further research is needed to determine impact of additional information from tissue samples and new developments in near patient testing on clinical outcomes and antibiotic stewardship.

Study Registration: NRES Ref:11/YH/0078;UKCRN ID:10440;ISRCTN:52608451

Strengths and limitations of this study

- The first appropriately powered prospective study to assess sample agreement.
- Investigates the relationship between baseline characteristics and agreement between types of specimen using multivariable modelling.
- Included a sub-study to investigate the potential clinical relevance of the different amount of information gleaned from tissue and swab results by conducting a blinded virtual clinic with clinicians determining whether the microbiology result would indicate a change in therapy.
- In this pragmatic study, presence of pathogens is based on those reported by the clinical microbiology laboratory and so may not reflect all organisms/isolates identified.

Introduction

Diabetes mellitus is now a worldwide pandemic, with the prevalence in the US now exceeding 14%[1]. In persons with diabetes, foot complications, most commonly ulceration, related to peripheral sensory and motor neuropathy and peripheral arterial disease [2,3] occurs in 15% to 25% during their lifetime. [4,5] At presentation, over half of diabetic foot ulcers (DFU) are clinically infected [6] and foot infection precedes approximately 80% of non-traumatic lower limb amputations [4,7,8].

Diagnosis of DFU infection includes clinical assessment for local inflammation and systemic inflammatory response.[9] Antibiotics are commonly initiated immediately (empiric treatment) and samples collected for identification of wound flora and sensitivities to tailor the antibiotic regimen, avoiding unnecessary broad-spectrum therapy and antibiotic resistance. [10-12]

For peer review only - http://bmjopen.bmj.com/site/about/guidelines.xhtml

Accurate culture results depend on collecting a sample which yields infected tissue but is not contaminated by colonizing flora. Sterile swabs are widely available, quick and easy to use, and can be collected by most types of healthcare personnel. Unfortunately, swabs typically sample superficial flora, including colonizers or contaminants, and because of their construction (cotton wool) may fail to grow anaerobic or fastidious pathogens. Recognizing these limitations, many laboratories offer only minimal processing of swabs. Alternatively tissue sampling may obtain tissue from the base of the wound, which requires more skill and time, but it may reveal more pathogens as well as being less susceptible to sampling contaminants. Despite exhortations to obtain tissue rather than swab samples from most authoritative guidelines [13, 14,15] many clinicians default to the swab method. Our previous systematic review identified few studies comparing swabs and tissue samples[16] with limitations including retrospective designs, inclusion of patients with various wounds, small cohorts and lack of contemporaneous sampling. Uncertainty was not resolved in subsequent studies. One[17] retrospectively reviewed 54 pairs of samples (from people with DFU but not all of whom had a wound infection) and reported that, swabs detected more species than tissue samples (finding additional species in 11.2% of cases, fewer species in 9.0% of cases, and completely different organisms in 6.7%). In a second study, 50 infected DFU patients were swabbed and had tissue samples taken (the latter considered a 'gold-standard').[18] Swabs had 100% sensitivity and specificity <20%. A third study compared tissue and swab specimens from 56 patients with an infected DFU.[19] They noted that swabs missed organisms identified from tissue, especially gram-negative bacteria, in patients with more severe infections.

A further limitation of the published literature is that there is an assumption that tissue samples are the 'gold-standard', but in practice this method may also miss wound flora and hence we proposed a study to assess agreement and disagreement between the two methods of collecting wound information, by considering pathogens as specified by UK Health Protection Agency [20].

For peer review only - http://bmjopen.bmj.com/site/about/guidelines.xhtml

Study Design

We assessed the agreement between tissue and swab samples in patients with a suspected infected DFU. A detailed description of the study methods is available.[21]

This was a multicentre, cross-sectional study of 400 people with diabetes mellitus (79% were female) in English primary and secondary care foot ulcer/diabetic clinics and hospital wards. Foot ulcer infection was diagnosed clinically based on signs and patients were eligible for enrolment if the clinician evaluating them planned to treat them with antibiotic therapy. Consenting patients had a swab and tissue sample taken from the same foot ulcer. These were processed and reported by the usual local clinical microbiology laboratory so that the information gathered would be relevant for clinical practice.

Co-primary endpoints were the extent of agreement between swab and tissue sampling for three microbiological parameters: 1) presence of isolates likely to be pathogens; 2) the number of bacterial pathogens reported per sample; and, 3) the prevalence among likely pathogens of resistance to antimicrobials.

In addition we investigated the clinical usefulness of the information provided by tissue versus swab samples, using a blinded clinical panel to interpret the microbiology results. Secondary objectives considered sampling-related adverse effects and the costs of sampling.

Eligibility Criteria

Patients were eligible if they had a diagnosis of diabetes mellitus (type 1 or 2), were at least 18 years old, with a suspected infected DFU, determined clinically. Patients were excluded if the treating clinician deemed it inappropriate to take a tissue or swab sample for any reason, the patient had previously been enrolled in the study, or they were unwilling or unable to provide informed consent.

For peer review only

Assessments

Sample Acquisition

We trained clinicians at all centres to collect samples using the UK HPA standards [20,22] via site visits and an e-Learning package that we developed for this purpose.[23] After wound cleansing and debridement (if required), a physician, nurse or podiatrist obtained the samples from the infected ulcer. Swab cultures were obtained by Levine's technique.[24] Tissue samples were collected by using a sterile dermal curette or scalpel and placed in the transport medium used locally. All samples were transferred to and processed by their local clinical microbiology laboratory.[20,22] Study samples received no special labelling or processing.

Clinical Assessments

Baseline data included a medical history and examination, including signs and symptoms of wound infection, previous treatments, and classifying the current status of the foot ulcer using PEDIS,[25] Wagner,[26] Clinical Signs and Symptoms Classification of Infection,[27] and a Pain scale after both swab and tissue sampling. Investigators reported adverse events associated with sample collection.

Centre differences questionnaire

Participating sites and microbiology laboratories completed a centre questionnaire regarding acquisition of samples, the transport, analysis, and reporting of samples by the laboratory, and their local antibiotic protocols, to evaluate the potential differences between centres.

Clinical Panel Review

We sought to compare the proportion of patients for whom the antibiotic regimen actually prescribed by the attending medical team was 'appropriate,' based on culture and sensitivity results of swab <u>or</u> tissue samples by sending 247 sample results along with a record of empirical antimicrobial regimen prescribed, to a panel of 13 clinicians clinically blinded to

For peer review only

whether the microbiology results were from a tissue or swab specimen. Clinicians were asked:

Question 1: 'Are there any pathogens identified in the lab report that are not covered by the prescribed antimicrobial regimen? (Yes/No)'

Question 2: 'If you answered 'yes' to question 1, would knowing this information lead you to prescribe an alternative antibiotic regimen for this patient? (Yes/No)'.

Sample Size

Our sample size was based on the primary outcome of reported 'presence or absence of a pathogen'. Our target sample size was 400, as we calculated that 399 patients would provide 80% power to detect a difference of ≥3% in the reported presence of a given pathogen, if overall prevalence was 10%, with 5% disagreement between the swab and tissue samples, using a two-sided McNemar's test at the 5% level of significance. This level of agreement would also provide a kappa statistic of 0.7. This calculation is based on less prevalent organisms, such as *Pseudomonas aeruginosa*[28] , hence the power was higher for more prevalent species.

STATISTICAL ANALYSIS

All tests of statistical significance were two-sided and based on the evaluable population, with p-values and 95% confidence intervals provided as appropriate.

Microbiology laboratories reported pathogens at a range of taxonomic levels which we grouped in order to report statistics meaningfully, i.e. by genus, species, etc. For pathogens with a prevalence >8% we generated cross-tabulations of reported presence in swab and tissue: overall percentage prevalence, agreement, and disagreement; unadjusted kappa for agreement; prevalence and bias adjusted kappa (PABAK) for agreement; prevalence difference (tissue – swab, and 95% CI); and McNemar's test for differences. As a number of scales were used by the participating laboratories to quantify the extent of growth of a

For peer review only - http://bmjopen.bmj.com/site/about/guidelines.xhtml

pathogen (for example, +/++/+++; +/++/+++/++++; scanty/light/moderate/heavy; scanty/+/++/+++; light, moderate, heavy), these were derived onto one three-point scale reported as +/++/+++. The derived data was used to tabulate the extent of bacterial growth (none, + to +++) and calculated weighted kappa statistics.

We pre-specified baseline factors to investigate their relevance in determining agreement between sample results including: type of ulcer (ischemic or neuro-ischaemic versus neuropathic); Wagner grade of ulcer (1-5); recent antimicrobial therapy; and wound duration. We generated an overall summary of pathogens,[29] and used univariable multinomial regression by centre to determine whether agreement was influenced by any of these factors.

Using univariable ordinal regression modelling we assessed the influence of baseline factors on the number of pathogens thus: tissue sampling (compared to swab) had 2 or more extra pathogens reported; tissue sampling had 1 extra pathogen reported; tissue and swab sampling had the same number of pathogens reported; swab sampling had 1 or more extra pathogens reported. In both regression analyses, centre was included as a random effect and multiple imputation was used to impute missing baseline factors.

For the clinical panel study of appropriateness of antibiotic treatment we summarised whether pathogens identified were or were not covered by the prescribed antimicrobial regimen. We also asked if, in the clinician's opinion a change in therapy was required. McNemar's test was used to identify whether one sampling method identified more patients requiring a change in therapy than the other.

RESULTS

Recruitment

Between 15th November 2011 and 15th May 2013 we screened 680 patients, and enrolled 401 patients from 25 centres. We excluded one patient whose consent was lost and 5 for

For peer review only

For peer review only - http://bmjopen.bmj.com/site/about/guidelines.xhtml

whom one or more sample was lost or misused, producing a full analysis set of 400 patients and evaluable population of 395 patients (Figure 1).

Demographics

Most participants were from clinics (79.8%) and were female (79%). They had a median age of 63 years (range 26 – 99), a median duration of diabetes of 16.8 years (range 9-23), and median duration of their index ulcer of 5.6 months (range 0.7 – 6.0). Before sampling, 60.3% had an antimicrobial dressing or agent on the suspected infected ulcer, and 46.8% had received systemic antibiotic therapy. After enrolment, 93.5% of participants received systemic antibiotics. (Table 1)

Microbiology results

Culture results yielded 79 different microbial isolates. Among the swab samples, there were no isolates reported from 20.0% and non-pathogenic isolates from 9.9%. Among tissue samples, there were no isolates reported in 10.1% and non-pathogen isolates from 3.8%. (Table 2)

The most frequently reported groups of pathogens were: gram-positive cocci (70.6%), gram-negative bacilli (36.7%), Enterobacteriaceae, including coliforms (26.6%), obligate anaerobes (23.8%), and gram-positive bacilli (11.1%). The most frequently reported pathogens were: *Staphylococcus aureus* (43.8%, of which 8.1% were methicillin-resistant), *Streptococcus* spp (16.7%), *Enterococcus* spp (14.9%), coagulase-negative *Staphylococcus* spp (12.2%), *Corynebacterium* spp (9.4%), and *Pseudomonas* spp (8.6%). All other genus and species level pathogens had a combined prevalence <6%. (Table 2)

Primary Endpoints

Summary of pathogens reported

For peer review only - http://bmjopen.bmj.com/site/about/guidelines.xhtml

For 58% of patients there was a difference in the pathogens reported by the two techniques. The swab reported additional pathogens to those in the tissue in 8.1%; the tissue reported additional pathogens to those in the swab in 36.7%; and, the tissue and swab samples reported different pathogens, with or without overlap, in 13.2%.

Reported presence of pathogens

For the majority of pathogens the reported prevalence was significantly higher from tissue samples than swab samples (McNemar's p-value < 0.01). Exceptions to this were *Staphylococcus aureus* and *Pseudomonas aeruginosa*, where we observed symmetrical disagreement, i.e. for the same number of people swabbing missed a pathogen picked up by tissue sampling, as there were pathogens missed by tissue sampling but detected by swabbing. A full cross-tabulation of the reported presence of all of these pathogens is shown in Table 2, with statistical analyses presented in Table 3.

Based on this summary, we undertook a univariable multinomial analysis to determine whether the outcome 'both swab and tissue report the same pathogens' was related to any of several potentially important patient baseline variables. (Table 4) None of the baseline factors examined had a significant effect on overall agreement.

Reported presence of antimicrobial resistance among likely pathogens

We investigated the reported presence of three common antimicrobial-resistant pathogens using two sampling methods. Methicillin-resistant *S. aureus* (MRSA) was reported in 6.8% of swabs and 7.8% of tissue samples, a difference (95% CI) of 1.0% (-0.2%, 2.8%), with McNemar's exact p-value=0.219). Vancomycin-resistant *Enterococcus* was reported in only 1 (0.3%) patient (detected by both swab and tissue).

Number of pathogens reported per sample

Swab and tissue samples had a median 1 pathogen per sample; a mean of 1.0 and 1.5 and maximum 4 and 6 pathogens, respectively. A greater proportion of swab samples reported

For peer review only - http://bmjopen.bmj.com/site/about/guidelines.xhtml

no pathogens compared to tissue samples (29.9% vs. 13.9% respectively). In 49.6% of patients the same number of pathogens was reported for the tissue and swab sample, for 41.5% at least one more pathogen was reported from the tissue than the swab sample, and for 8.9% of there was at least one more pathogen reported from the swab than the tissue sample.

The univariable ordinal analysis found that patients' tissue samples were reported to have 2 or more additional pathogens significantly more often if their ulcer was present for ≥56 days than if it was present <56 days (OR 1.56, 95% CI 1.05, 2.33, p=0.024).

Clinical Panel Review

For 73.3% cases there was agreement on the requirement for a change in therapy between swab and tissue samples (kappa 0.45: 95%CI 0.34, 0.56), which is moderate agreement. In 17.8% of cases the clinician indicated that the tissue sample results would lead to a recommendation of change in therapy, when the swab sample did not indicate a need for change. Likewise, in 8.9% of results the clinician indicated that the swab result would lead to a change in therapy whereas the tissue sample did not (increase of 8.9% 95% CI (2.65, 15.3%)).

Adverse Events

"Bleeding of concern" during sample collection was reported in 30 (7.6%) patients: attributed to swabbing in 6 (1.5%) and to tissue sampling in 27 patients (6.8%). Different levels of pain after either swab or tissue sampling was reported by 10.5% (42/400) of patients. Of these 5 (1.3%) patients reported worse pain after swabbing compared to tissue sampling, and 37 (9.3%) patients reported worse pain after tissue sampling compared to swabbing.

Centre differences

For peer review only

We received Questionnaires from 22 centres: one site used a dermal curette to collect tissue samples and others used a scalpel. There were no differences in time taken for swab and tissue samples to reach the lab from clinic and no difference in the time taken from receipt of samples to processing: 4/17 (23.5%) reported a slightly more urgent processing of tissue samples.

A Gram-stained smear of the specimen was more common for tissue than swab samples, 9/19 (47.4%) laboratories did this for tissue only; 3/19 (15.8%) did it for both samples; whilst 6/19 (31.6%) did not perform Gram staining (available only on request in 1 laboratory). More than half the laboratories, 10/18 (55.6%), reported all isolates from a tissue sample but tailored reports from a swab according to clinical details and significance. Centre differences were apparent in the multinomial and ordinal regression analysis where its inclusion improved the fit of both models ($p<0.001$).

Only two laboratories provided data on the cost of processing hence analysis was not possible.

Discussion

To our knowledge this is the largest comparison of the two main methods of sampling, the first to report detailed data on paired samples for each pathogen and the first to examine the relationship between baseline characteristics and agreement between types of specimen using multivariable modelling. This study has several additional strengths: All centres received update training on swab and tissue sampling to minimise between sample differences. We prospectively enrolled a large number of patients at a large number of similar clinical sites using a well-defined protocol and obtaining contemporaneous dual specimens. The study also has high external validity, as we had minimal exclusion criteria,

For peer review only - http://bmjopen.bmj.com/site/about/guidelines.xhtml

we recruited patients in usual practice settings, members of the attending clinical teams obtained the samples, and the local laboratories processed the specimens.

We found that tissue sampling had a higher yield than swab specimens, hence it provided more information on wound flora. While tissue sampling was both more sensitive and specific than swabs, both techniques missed some organisms. Thus, to some degree they provide complementary information hence both techniques may be useful. The differences in the results of the two sampling techniques may be due to: the tissue specimen providing a greater yield of organisms at collection or a lower rate of organism death during transport; or, to the way the microbiology laboratory handled or reported the culture results. In settings where obtaining wound specimens by swab remains the preferred method, we suggest examining these issues to try to increase the yield of wound cultures. It is important to note that there is no validated diagnostic threshold for diagnosing clinical infection in diabetic foot wounds. Thus, the substantial proportion of samples in our study that reported no pathogens may reflect false positive diagnoses of infection.[30] Alternatively, this may be related to: the sampling technique; poor collection methods (e.g., not expressing fluid in Levine's technique [24]); transport media that fail to maintain the viability of swab pathogens; the high percentage of patients who used antimicrobial agents before the specimen was taken; or, a decision by the microbiologist to report only pathogens that they deemed necessary to report.

A key clinical issue is how much, and what type of, information on ulcer flora is useful for clinicians managing patients with an infected diabetic foot ulcer. While clinicians want to optimally target their antibiotic therapy, providing microbiology reports with lists of many organisms, including non-pathogenic or unusual isolates present in low numbers, may confuse rather than aid decision-making. We do not know if treatment based upon a more detailed microbiogram is helpful in selecting an antimicrobial regimen that increases the

For peer review only

likelihood of, or time to, resolution of infection, or the prevention of treatment associated antibiotic resistance.

We found that more clinicians presented with tissue than swab sample microbiology reports (while blinded to the type of specimen) recommended a change in antibiotic therapy, suggesting that the additional information tissue specimens provide could lead to more tailored antimicrobial regimens. It is not clear whether this is theoretical finding would be confirmed in practice.

Given the global emergency associated with antibiotic-resistance related to over-use of this precious resource, we wish to be cautious about recommending a technique that may lead to unnecessarily broad-spectrum prescribing. Furthermore, the bacterial flora in the wound at the time of sampling presentation may differ from those present days later after empiric antibiotic therapy, when culture results are reported, potentially reducing the utility of this information.

Previous reports comparing swab to tissue specimens have been small, single-centre studies, for example in a retrospective study of 89 concomitantly obtained pairs of samples from 54 patients with diabetic foot ulcers (87% clinically infected), Mutluogou [16] found culture results of superficial swabs did not correlate well with those obtained from deep tissue, however they summarised results in terms of predictive value for infection in spite of there being no 'gold-standard' for this. In 50 patients with an infected diabetic foot ulcer, Demetriou et al [17] compared a tissue culture against a swab specimen culture and found that reports agreed in only 50% of patients. In a further study of 56 patients with diabetic foot infection, grouped according to the PEDIS grading system, Huang et al[19] found swab culturing identified all micro-organisms isolated from the corresponding deep tissue culture in 90% of grade 2 wounds, and in 41.4% and 41.2% for grade 3 and 4 wounds respectively.

We believe our results demonstrate increased sensitivity of tissue compared to swab specimens. What remains uncertain is whether this increased information results in more appropriate prescribing and/or better outcomes including resolution of infection and healing.

For peer review only

Therefore, further research is needed to determine the impact of additional information from tissue samples and on new developments in near patient testing on clinical outcomes and antibiotic stewardship. This will then inform the most appropriate method of obtaining specimens from diabetic foot ulcers.

END

For peer review only

Funding: This work was supported by the National Institute for Health Research (NIHR) Health Technology Assessment (HTA) programme (project number 09/75/01).

Disclaimer: The views and opinions expressed herein are those of the authors and do not necessarily reflect those of the HTA programme, NIHR, NHS or the Department of Health.

Competing interests: All authors declare they have no competing interests

Ethics Approval: CODIFI received ethical approval from the Sheffield NRES Committee (Ref: 11/YH/0078) and each enrolling site obtained local ethical approval prior to commencing recruitment.

Data Sharing statement: Requests for data should be made to the corresponding author

Authors' Contributions: Professor E. Andrea Nelson (Professor of Wound Healing) was the chief investigator for the study and is the gaurantor. She initiated the study, led the grant application development and the study team, and was involved in drafting and revising this paper critically for intellectual content. Miss Alexandra Wright-Hughes (Senior Statistician) was a member of the study team, contributed to the drafting of the statistical analysis plan, undertook the statistical analyses, drafted the statistical results and contributed to the drafting and revising paper for intellectual content. Dr Michael Backhouse (NIHR Research Fellow, Podiatrist) was the first clinical study coordinator, and a member of the study team, initiated sites for recruitment, contributed to the drafting of the study report and revised it for intellectual content. Professor Benjamin A. Lipsky (Professor of Medicine) was a co-applicant on the study grant, a member of the study team, contributed to the drafting of this paper and revised it for intellectual content. Professor Jane Nixon (Professor of tissue viability) was a co-applicant on the study grant, was a member of the study team, the lead for the Clinical Trials Unit activity and responsible for these aspects, she contributed to the drafting of the manuscript and reviewed it for intellectual content. Dr Moninder Bhogal (Senior Trial Coordinator) was the trials unit study coordinator, and a member of the study team, he was responsible for ethics and governance applications and coordinated the study

For peer review only

team, he contributed to the drafting of the study report and revised it for intellectual content. Miss Catherine Reynolds (Senior Data Manager), was the data manager and a member of the study team, responsible for data quality, contributed to the drafting of the study report and revised it for intellectual content. Miss Sarah Brown (Principal Statistician) the supervising statistician, was a co-applicant on the study grant, and a member of the study team, contributed to the drafting of the statistical analysis plan, oversaw the statistical analyses and preparation of the statistical results, she contributed to the drafting of the manuscript and reviewed it for intellectual content.

For peer review only

Acknowledgements

We thank members of the study steering committee (Prof J Deeks, Prof R Cooper, Prof R Gadsby, Prof AM Keenan, Mrs C Thomas).

The CODIFI collaborators were EA Nelson, J Nixon, S Brown, J Gray, J Firth, C Dowson, E Jude, T Dickie, C Amery, G Sykes, P Vowden, M Edmonds

For peer review only

References

1. Menke A CS, Geiss L, Cowie CC. Prevalence of and Trends in Diabetes Among Adults in the United States, 1988-2012. Journal of the American Medical Association 2015; 314(10): 1021-9.

2. Vinik AI, Maser RE, Mitchell BD, Freeman R. Diabetic autonomic neuropathy. Diabetes care 2003; 26(5): 1553.

3. Richard JL, Lavigne JP, Sotto A. Diabetes and foot infection: more than double trouble. Diabetes/Metabolism Research and Reviews 2012; 28: 46-53.

4. Singh N, Armstrong DG, Lipsky BA. Preventing foot ulcers in patients with diabetes. JAMA: the journal of the American Medical Association 2005; 293(2): 217.

5. Lavery L, Armstrong D, Wunderlich R, Tredwell J, Boulton A. Diabetic foot syndrome: evaluating the prevalence and incidence of foot pathology in Mexican Americans and non-Hispanic whites from a diabetes disease management cohort. Diabetes care 2003; 26(5): 1435.

6. Prompers L, Huijberts M, Schaper N, et al. Resource utilisation and costs associated with the treatment of diabetic foot ulcers. Prospective data from the Eurodiale Study. Diabetologia 2008; 51(10): 1826-34.

7. Reiber G. The epidemiology of diabetic foot problems. Diabetic medicine: a journal of the British Diabetic Association 1996; 13: S6.

8. Armstrong DG, Lavery LA, Quebedeaux TL, Walker SC. Surgical morbidity and the risk of amputation due to infected puncture wounds in diabetic versus nondiabetic adults. Journal of the American Podiatric Medical Association 1997; 87(7): 321-6.

9. B. A. Lipsky JA-S, M. Diggle, J. Embil, S. Kono, L. Lavery, É. Senneville, V. Urbancic-Rovan, S. Van Asten6,9, E. J. G. Peters, on behalf of the International Working Group on the Diabetic Foot (IWGDF) IWGDF Guidance on the diagnosis and management of foot infections in persons with diabetes, Prepared by the IWGDF Working Group on Foot Infections

2015. http://iwgdf.org/guidelines/guidance-on-infection/

10. Lipsky B. Empirical therapy for diabetic foot infections: are there clinical clues to guide antibiotic selection? Clinical microbiology and infection 2007; 13(4): 351-3.

11. Lipsky BA. A report from the international consensus on diagnosing and treating the infected diabetic foot. Diabetes/Metabolism Research and Reviews 2004; 20(S1): S68-S77.

12. Lipsky BA, Berendt AR, Deery HG, et al. Diagnosis and treatment of diabetic foot infections. Clinical Infectious Diseases 2004; 39(7): 885.

13. Lipsky BA, Berendt AR, Cornia PB, et al. 2012 Infectious Diseases Society of America clinical practice guideline for the diagnosis and treatment of diabetic foot infections. Clinical infectious diseases : an official publication of the Infectious Diseases Society of America 2012; 54(12): e132-73.

For peer review only

14. Lipsky BA A-SJ, Diggle M, Embil J, Kono S, Lavery L, Senneville É, Urbančič-Rovan V, Van Asten S; International Working Group on the Diabetic Foot. IWGDF guidance on the diagnosis and management of foot infections in persons with diabetes. Diabetes Metab Res Rev 2016; 32(Suppl 1): 45-74.

15. Excellence NIfHaC. Diabetic foot problems: prevention and management [NG19]. In: Excellence NIfHaC, editor. London; 2016.

16. Nelson EA, O'Meara S, Craig D, et al. A series of systematic reviews to inform a decision analysis for sampling and treating infected diabetic foot ulcers. Health technology assessment 2006; 10(12): iii-iv, ix-x, 1-221.

17. Mutluoglu M, Uzun G, Turhan V, Gorenek L, Ay H, Lipsky BA. How reliable are cultures of specimens from superficial swabs compared with those of deep tissue in patients with diabetic foot ulcers? Journal of diabetes and its complications 2012; 26(3): 225-9.

18. Demetriou M, Papanas N, Panopoulou M, Papatheodorou K, Bounovas A, Maltezos E. Tissue and swab culture in diabetic foot infections: neuropathic versus neuroischemic ulcers. The international journal of lower extremity wounds 2013; 12(2): 87-93.

19. Huang Y CY, Zou M, Luo X, Jiang Y, Xue Y, Gao F. A Comparison of Tissue versus Swab Culturing of Infected Diabetic Foot Wounds. Int J Endocrinol 2016; 8198714.

20. HPA. Investigation of skin, superficial and non-surgical wound swabs. In: Agency HP, editor. London; 2009.

21. Nelson EA, Backhouse MR, Bhogal MS, et al. Concordance in diabetic foot ulcer infection. BMJ open 2013; 3(1).

22. HPA. Investigation of tissues and biopsies. In: Agency HP, editor. London; 2009.

23. Randell R, Backhouse MR, Nelson EA. Videoconferencing for site initiations in clinical studies: Mixed methods evaluation of usability, acceptability, and impact on recruitment. Informatics for Health and Social Care 2015: 1-11.

24. Levine NS, Lindberg RB, Mason AD, Jr., Pruitt BA, Jr. The quantitative swab culture and smear: A quick, simple method for determining the number of viable aerobic bacteria on open wounds. The Journal of trauma 1976; 16(2): 89-94.

25. Schaper N. Diabetic foot ulcer classification system for research purposes: a progress report on criteria for including patients in research studies. Diabetes/Metabolism Research and Reviews 2004; 20(S1): S90-S5.

26. Wagner Jr F. The diabetic foot. Orthopedics 1987; 10(1): 163-72.

27. Gardner S, Frantz R, Troia C, et al. A tool to assess clinical signs and symptoms of localized infection in chronic wounds: development and reliability. Ostomy/Wound Management 2001; 47(1): 40.

For peer review only

28. Pellizzer G, Strazzabosco M, Presi S, et al. Deep tissue biopsy vs. superficial swab culture monitoring in the microbiological assessment of limb-threatening diabetic foot infection. Diabetic medicine 2001; 18(10): 822-7.

29. Slater R, Lazarovitch T, Boldur I, et al. Swab cultures accurately identify bacterial pathogens in diabetic foot wounds not involving bone. Diabetic medicine 2004; 21(7): 705-9.

30. Edmonds M, Foster A. The use of antibiotics in the diabetic foot. Am J Surg 2004; 187(5A): 25s-8s.

For peer review only

For peer review only - http://bmjopen.bmj.com/site/about/guidelines.xhtml

Table 1 Baseline characteristics of enrolled patients

Characteristic	Clinical Values	Full Analysis Set (n=400)
**Age (years)**	Mean (SD)	63·1 (13·3)
	Median, range and (IQR)	63·0 [26-99] (54·0, 73·0)
**Sex**	Male	316 (79·0%)
	Female	84 (21·0%)
**Ethnicity**	White	377 (94·3%)
	Other	23 (5·7%)
**Site of recruitment**	Hospital ward	53 (13·3%)
	Outpatient clinic	319 (79·8%)
	Community clinic	28 (7·0%)
**Diabetes type**	Type 1	58 (14·5%)
	Type 2	342 (85·5%)
**Duration of diabetes (years)**	N Missing	3
	Mean (SD)	16·8 (11·0)
	Median, range and (IQR)	15·0 [0·04-57] (9·0, 23·0)
**Diabetes treatment details**	Oral hypoglycaemic agent	107 (27·8%)
	Insulin	168 (43·6%)
	Oral hypoglycaemic agent & Insulin	109 (28·3%)
	Other	1 (0·3%)
	None	15 (3·8%)
**Number of foot ulcers**	1	268 (67·0%)
	≥2	132 (33·0%)
**Duration of index ulcer (months)**	N Missing	4
	Mean (SD)	5·58 (12·28)
	Median, [range] and (IQR)	1·84 [0·1-144·0] (0·69, 6·00)
**Aetiology of index ulcer**	Ischemic	14 (3·5%)
	Neuropathic	202 (50·5%)
	Ischemic & Neuropathic	182 (45·5%)
	Missing	2 (0·5%)
**Antimicrobial dressing on ulcer**	Yes	241 (60·3%)
	No	154 (38·5%)
	Missing	5 (1·3%)
**Patient already on systemic antibiotics**	Yes	187 (46·8%)
	No	194 (48·5%)
	Missing	19 (4·8%)
**Patient on antibiotics immediately post sampling**	Yes	374 (93·5%)
	No	26 (6·5%)
**Grade (Wagner scale)[a]**	Grade 1	136 (34·0%)
	Grade 2	134 (33·5%)
	Grade 3	122 (30·5%)
	Grade 4	7 (1·8%)
	Grade 5	1 (0·3%)

[a] Grade 1 - Superficial diabetic ulcer (partial or full thickness); Grade 2 – Ulcer extension ligament, tendon, joint capsule, or deep fascia without abscess or osteomyelitis; Grade 3 – Deep ulcer with abscess, osteomyelitis or joint sepsis; Grade 4 – Gangrene localized to portion of forefoot or heel; Grade 5 – Extensive gangrenous involvement of the entire foot·

For peer review only

Table 2 Cross tabulation of reported presence of at least one pathogen and pathogens with >8% prevalence in order of taxonomic rank and prevalence

**Pathogen (Overall prevalence)**			**Tissue Results**	**Tissue Results**	
			Not reported	***Reported***	***Total***
**At least one pathogen (88·1%)**	***Swab***	***Not reported***	47 (11·9%)	71 (18·0%)	118 (29·9%)
	Swab	***Reported***	8 (2·0%)	269 (68·1%)	277 (70·1%)
		Total	55 (13·9%)	340 (86·1%)	395 (100·0%)
**Gram-positive cocci (70·6%)**	***Swab***	***Not reported***	116 (29·4%)	68 (17·2%)	184 (46·6%)
	Swab	***Reported***	14 (3·5%)	197 (49·9%)	211 (53·4%)
		Total	130 (32·9%)	265 (67·1%)	395 (100·0%)
**Gram-negative bacilli (36·7%)**	***Swab***	***Not reported***	250 (63·3%)	49 (12·4%)	299 (75·7%)
	Swab	***Reported***	12 (3·0%)	84 (21·3%)	96 (24·3%)
		Total	262 (63·3%)	133 (33·7%)	395 (100·0%)
**Enterobacteriacea (including coliforms) (26·6%)**	***Swab***	***Not reported***	290 (73·4%)	37 (9·4%)	327 (82·8%)
	Swab	***Reported***	14 (3·5%)	54 (13·7%)	68 (17·2%)
		Total	304 (77·0%)	91 (23·0%)	395 (100·0%)
**Obligate anaerobes (23·8%)**	***Swab***	***Not reported***	301 (76·2%)	46 (11·6%)	347 (87·8%)
	Swab	***Reported***	19 (4·8%)	29 (7·3%)	48 (12·2%)
		Total	320 (81·0%)	75 (19·0%)	395 (100·0%)
**Gram-positive bacilli (11·1%)**	***Swab***	***Not Reported***	351 (88·9%)	40 (10·1%)	391 (99·0%)
	Swab	***Present***	1 (0·3%)	3 (0·8%)	4 (1·0%)
		Total	352 (89·1%)	43 (10·9%)	395 (100·0%)
***Streptococcus* spp (16·7%)**	***Swab***	***Not reported***	329 (83·3%)	18 (4·6%)	347 (87·8%)
	Swab	***Reported***	5 (1·3%)	43 (10·9%)	48 (12·2%)
		Total	334 (84·6%)	61 (15·4%)	395 (100·0%)
***Enterococcus* spp (excluding VRE) (14·9%)**	***Swab***	***Not reported***	336 (85·1%)	34 (8·6%)	370 (93·7%)
	Swab	***Reported***	6 (1·5%)	19 (4·8%)	25 (6·3%)
		Total	342 (86·6%)	53 (13·4%)	395 (100·0%)
**Coagulase-negative *Staphylococcus* spp (12·2%)**	***Swab***	***Not reported***	347 (87·8%)	39 (9·9%)	386 (97·7%)
	Swab	***Reported***	1 (0·3%)	8 (2·0%)	9 (2·3%)
		Total	348 (88·1%)	47 (11·9%)	395 (100·0%)
***Corynebacterium* spp (9·4%)**	***Swab***	***Not reported***	358 (90·6%)	33 (8·4%)	391 (99·0%)
	Swab	***Reported***	1 (0·3%)	3 (0·8%)	4 (1·0%)
		Total	359 (90·9%)	36 (9·1%)	395 (100·0%)
***Pseudomonas aeruginosa* (8·6%)**	***Swab***	***Not reported***	361 (91·4%)	8 (2·0%)	369 (93·4%)
	Swab	***Reported***	8 (2·0%)	18 (4·6%)	26 (6·6%)
		Total	369 (93·4%)	26 (6·6%)	395 (100·0%)
***Staphylococcus aureus* (excluding MRSA) (35·7%)**	***Swab***	***Not reported***	254 (64·3%)	16 (4·1%)	270 (68·4%)
	Swab	***Reported***	16 (4·1%)	109 (27·6%)	125 (31·6%)
		Total	270 (68·4%)	125 (31·6%)	395 (100·0%)
**Methicillin-resistant *S· aureus* (8·1%)**	***Swab***	***Not reported***	363 (91·9%)	5 (1·3%)	368 (93·2%)
	Swab	***Reported***	1 (0·3%)	26 (6·6%)	27 (6·8%)
		Total	364 (92·2%)	31 (7·8%)	395 (100·0%)

For peer review only

Abbreviations: VRE= vancomycin-resistant enterococcus, MRSA= methicillin-resistant *Staphylococcus aureus*

For peer review only

Table 3 Summary of agreement and disagreement statistics for most prevalent pathogens and the report of at least one pathogen

	Overall prevalence	Overall disagreement	Difference (95% CI)*	McNemar's P Value	Overall agreement	Unadjusted Kappa (95% CI)	PABAK
**At least one pathogen**	88·1%	20·0%	15·9% (11·8%, 20·1%)	<0·0001	80·0%	0·44 (0·34, 0·53)	0·60
**Gram-positive cocci**	70·6%	20·8%	13·7% (9·4%, 18·0%)	<0·0001	79·2%	0·57 (0·50, 0·65)	0·58
**Gram-negative bacilli**	36·7%	15·4%	9·4% (5·6%, 13·1%)	<0·0001	84·6%	0·63 (0·55,0·71)	0·69
**Enterobactereacea (Including coliforms)**	26·6%	12·9%	5·8% (2·3%,9·3%)	0·0013	87·1%	0·60 (0·50,0·70)	0·74
**Obligate anaerobes**	23·8%	16·5%	6·8% (2·9%, 10·8%)	0·0008	83·5%	0·38 (0·26,0·50)	0·67
**Gram-positive bacilli**	11·1%	10·4%	9·9% (6·9%, 13·5%)	<0·0001**	89·6%	0·11 (-0·01,0·23)	0·79
Streptococcus spp	16·7%	5·8%	3·3% (0·9%, 5·6%)	0·0067	94·2%	0·76 (0·66,0·85)	0·88
***Enterococcus* spp (exc· VRE)**	14·9%	10·1%	7·1% (4·0%, 10·1%)	<0·0001	89·9%	0·44 (0·30,0·58)	0·80
**Coagulase-negative *Staphylococcus***	12·2%	10·1%	9·6% (6·7%, 12·9%)	<0·0001**	89·9%	0·26 (0·11,0·41)	0·80
Corynebacterium	9·4%	8·6%	8·1% (5·4%, 11·2%)	<0·0001**	91·4%	0·13 (-0·01,0·28)	0·83
Pseudomonas aeruginiosa	8·6%	4·1%	0·0% (-2·0%, 2·0%)	1·0000	95·9%	0·67 (0·52,0·82)	0·92
***Staphylococcus aureus* (exc· MRSA)**	35·7%	8·1%	0·0% (-2·8%, 2·8%)	1·0000	91·9%	0·81 (0·75,0·87)	0·84

*Tissue – swab, **exact p-value / CI

For peer review only

Table 4 Multinomial and ordinal regression models for individually fitted baseline factors

		Odds Ratio (95% CI)	AIC	Reduction in -2LogL	DF	P-value
**Multinomial Summary of Isolates**	Both swab and tissue report the same pathogens vs:					
**Null Model**			941·29			
**Ulcer Type**[1]						
Any Ischemia vs Neuropathic only	Swab > pathogens compared to the tissue	1·03(0·48,2·20)				
Any Ischemia vs Neuropathic only	Tissue > pathogens compared to the swab	0·86(0·53,1·40)	945·72	1·570	3	0·67
Any Ischemia vs Neuropathic only	Swab and tissue report totally different pathogens	0·68(0·35,1·31)				
**Ulcer Grade**						
Grade 2 vs Grade 1	Swab > pathogens compared to the tissue	0·68(0·26,1·78)				
Grade 2 vs Grade 1	Tissue > pathogens compared to the swab	1·08(0·60,1·93)				
Grade 2 vs Grade 1	Swab and tissue report totally different pathogens	1·14(0·51,2·54)	949·16	4·125	6	0·66
Grade 3/4/5 v Grade 1	Swab> pathogens compared to the tissue	1·28(0·52,3·11)				
Grade 3/4/5 v Grade 1	Tissue > pathogens compared to the swab	1·60(0·87,2·95)				
Grade 3/4/5 v Grade 1	Swab and tissue report totally different pathogens	1·55(0·69,3·45)				
**Previous antibiotic therapy**[1]						
Yes vs No	Swab > pathogens compared to the tissue	0·80(0·36,1·80)				
Yes vs No	Tissue > pathogens compared to the swab	1·14(0·69,1·89)	946·28	1·005	3	0·80
Yes vs No	Swab and tissue report totally different pathogens	1·10(0·56,2·16)				
**Antimicrobial Dressing**[1]						
Yes vs No	Swab > pathogens compared to the tissue	1·13(0·51,2·51)				
Yes vs No	Tissue > pathogens compared to the swab	0·69(0·40,1·19)	943·44	3·850	3	0·28
Yes vs No	Swab and tissue report totally different pathogens	1·38(0·66,2·89)				

For peer review only - http://bmjopen.bmj.com/site/about/guidelines.xhtml

**Wound Duration (Median split)** [1]						
<56 days vs >=56 days	Swab > pathogens compared to the tissue	0·94 (0·43, 2·04)				
<56 days vs >=56 days	Tissue > pathogens compared to the swab	1·75 (1·08, 2·86)*	941·48	5·802	3	0·12
<56 days vs >=56 days	Swab and tissue report totally different pathogens	1·14 (0·59, 2·17)				
**Log Wound Duration (Continuous)** [1]						
	Swab > pathogens compared to the tissue	0·95(0·72,1·25)				
	Tissue > pathogens compared to the swab	0·88(0·74,1·04)	944·97	2·318	3	0·51
	Swab and tissue report totally different pathogens	0·93(0·74,1·18)				
**Ordinal Summary of Isolates**						
**Null Model**			917·72			
**Ulcer Type[1]:**Any Ischaemia vs Neuropathic only		0·90(0·61,1·33)	919·45	0·271	1	0·60
**Ulcer Grade**			920·16	1·559	2	0·46
Grade 2 vs Grade 1		1·33(0·82,2·15)				
Grade 3, 4, or 5 vs Grade 1		1·27(0·78,2·07)				
**Previous antibiotic therapy[1]:**Yes vs No		1·25(0·81,1·91)	918·56	1·154	1	0·28
**Antimicrobial Dressing[1]:** Yes vs No		0·76(0·49,1·18)	918·16	1·553	1	0·21
**Wound Duration (Median split)[1]:** <56 days vs >=56 days		1·56(1·05,2·33)	914·62	5·097	1	0·02**
**Log Wound Duration (Continuous)[1]**		0·92(0·80,1·05)	918·15	1·571	1	0·21

Note: Based on the evaluable population N=395 / [a]Smaller is better / [1] factors with missing data from the 28 (7·1%) patients with at least one missing data item / **significant at the 5% level

For peer review only - http://bmjopen.bmj.com/site/about/guidelines.xhtml

Figure

Fig 1 Study Recruitment Diagram

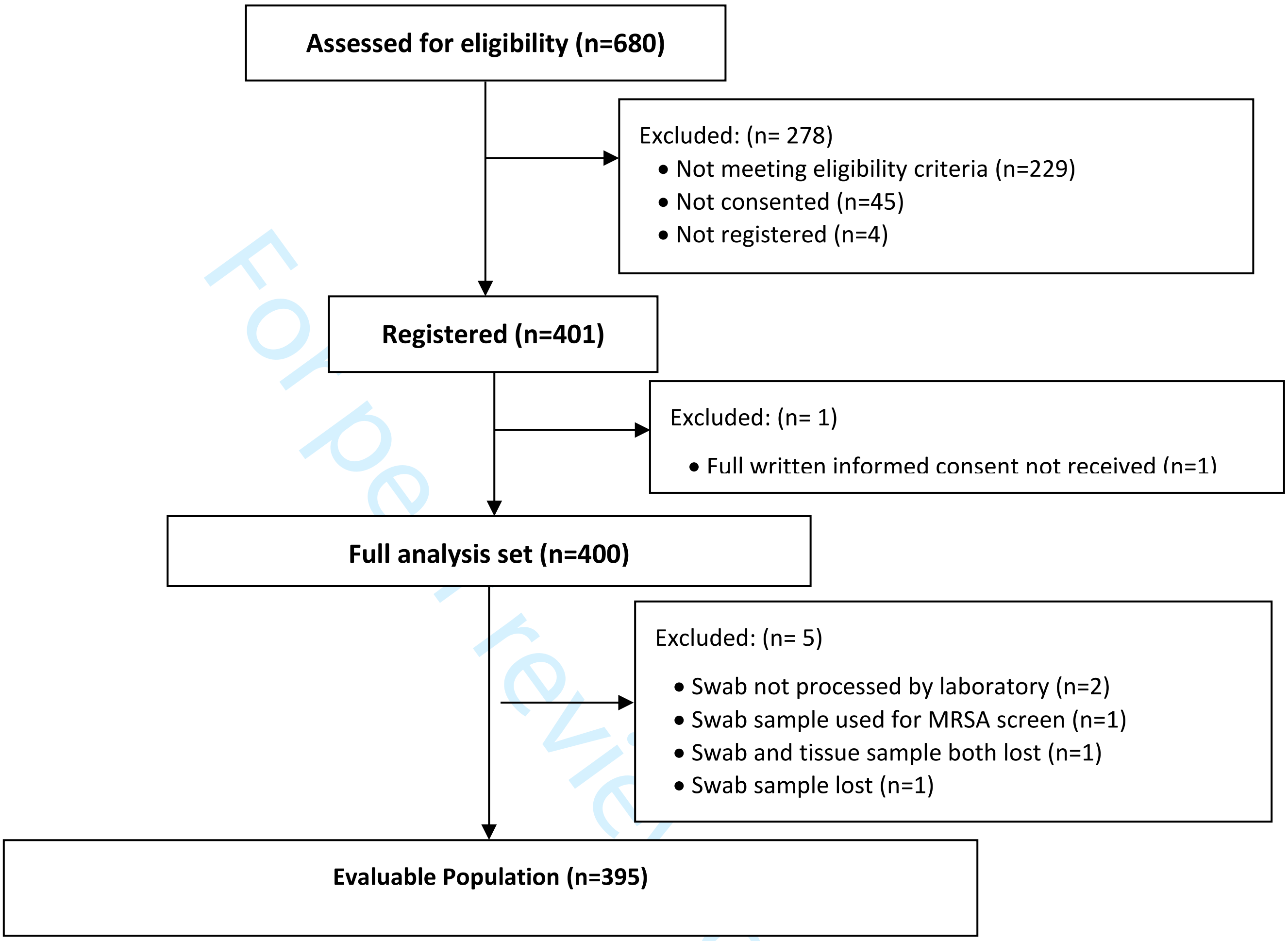

For peer review only

STROBE 2007 (v4) Statement—Checklist of items that should be included in reports of *cross-sectional studies*

Section/Topic	Item #	Recommendation	Reported on page #
**Title and abstract**	1	(*a*) Indicate the study's design with a commonly used term in the title or the abstract	1
		(*b*) Provide in the abstract an informative and balanced summary of what was done and what was found	2
**Introduction**			
Background/rationale	2	Explain the scientific background and rationale for the investigation being reported	4
Objectives	3	State specific objectives, including any prespecified hypotheses	5
**Methods**			
Study design	4	Present key elements of study design early in the paper	5
Setting	5	Describe the setting, locations, and relevant dates, including periods of recruitment, exposure, follow-up, and data collection	5; 9
Participants	6	(*a*) Give the eligibility criteria, and the sources and methods of selection of participants	6
Variables	7	Clearly define all outcomes, exposures, predictors, potential confounders, and effect modifiers. Give diagnostic criteria, if applicable	5, 6
Data sources/ measurement	8*	For each variable of interest, give sources of data and details of methods of assessment (measurement). Describe comparability of assessment methods if there is more than one group	5-7
Bias	9	Describe any efforts to address potential sources of bias	*5*
Study size	10	Explain how the study size was arrived at	7
Quantitative variables	11	Explain how quantitative variables were handled in the analyses. If applicable, describe which groupings were chosen and why	7-8
Statistical methods	12	(*a*) Describe all statistical methods, including those used to control for confounding	8-9
		(*b*) Describe any methods used to examine subgroups and interactions	8
		(*c*) Explain how missing data were addressed	8
		(*d*) If applicable, describe analytical methods taking account of sampling strategy	8
		(*e*) Describe any sensitivity analyses	N/A
**Results**			

For peer review only

Participants	13*	(a) Report numbers of individuals at each stage of study—eg numbers potentially eligible, examined for eligibility, confirmed eligible, included in the study, completing follow-up, and analysed	9
		(b) Give reasons for non-participation at each stage	9; figure 1
		(c) Consider use of a flow diagram	Figure 1
Descriptive data	14*	(a) Give characteristics of study participants (eg demographic, clinical, social) and information on exposures and potential confounders	9
		(b) Indicate number of participants with missing data for each variable of interest	22 (Table 1)
Outcome data	15*	Report numbers of outcome events or summary measures	10-12
Main results	16	(*a*) Give unadjusted estimates and, if applicable, confounder-adjusted estimates and their precision (eg, 95% confidence interval). Make clear which confounders were adjusted for and why they were included	10-11; 26 (Table 4)
		(*b*) Report category boundaries when continuous variables were categorized	11; 26
		(*c*) If relevant, consider translating estimates of relative risk into absolute risk for a meaningful time period	N/A
Other analyses	17	Report other analyses done—eg analyses of subgroups and interactions, and sensitivity analyses	11-12
**Discussion**			
Key results	18	Summarise key results with reference to study objectives	13
Limitations	19	Discuss limitations of the study, taking into account sources of potential bias or imprecision. Discuss both direction and magnitude of any potential bias	13
Interpretation	20	Give a cautious overall interpretation of results considering objectives, limitations, multiplicity of analyses, results from similar studies, and other relevant evidence	14-15
Generalisability	21	Discuss the generalisability (external validity) of the study results	13
**Other information**			
Funding	22	Give the source of funding and the role of the funders for the present study and, if applicable, for the original study on which the present article is based	16

*Give information separately for cases and controls in case-control studies and, if applicable, for exposed and unexposed groups in cohort and cross-sectional studies.

Note: An Explanation and Elaboration article discusses each checklist item and gives methodological background and published examples of transparent reporting. The STROBE checklist is best used in conjunction with this article (freely available on the Web sites of PLoS Medicine at http://www.plosmedicine.org/, Annals of Internal Medicine at http://www.annals.org/, and Epidemiology at http://www.epidem.com/). Information on the STROBE Initiative is available at www.strobe-statement.org.

For peer review only

BMJ Open

CODIFI (Concordance In Diabetic Foot Ulcer Infection) - a cross-sectional study of wound swabbing and tissue sampling in infected diabetic foot ulcers in England

Journal:	*BMJ Open*
Manuscript ID	bmjopen-2017-019437.R1
Article Type:	Research
Date Submitted by the Author:	24-Nov-2017

Complete List of Authors:	Nelson, E. Andrea; University of Leeds, School of Healthcare<br>Wright-Hughes, Alexandra; University of Leeds, Clinical Trials Research Unit<br>Backhouse, Michael; University of Leeds, Leeds Institute of Rheumatic and Musculoskeletal Medicine<br>Lipsky, BA; University of Oxford, Division of Medical Sciences<br>Nixon, Jane; University of Leeds, Clinical Trials Research Unit<br>Bhogal, Moninder; University of Leeds, School of Biomedical Sciences<br>Reynolds, Catherine; University of Leeds, Clinical Trials Research Unit<br>Brown, Sarah; University of Leeds, Clinical Trials Research Unit

**Primary Subject Heading**:	Diabetes and endocrinology
Secondary Subject Heading:	Research methods
Keywords:	diabetic foot infection, agreement, wound swab sample, tissue sample, diabetic foot ulcers

SCHOLARONE™
Manuscripts

CODIFI (Concordance In Diabetic Foot Ulcer Infection) - a cross-sectional study of wound swabbing and tissue sampling in infected diabetic foot ulcers in England

E. Andrea Nelson[1] E.A.Nelson@leeds.ac.uk

Alexandra Wright-Hughes[2] A.Wright-Hughes@leeds.ac.uk

Michael R Backhouse[3] M.R.Backhouse@leeds.ac.uk

Benjamin A. Lipsky[4] balipsky@uw.edu

Jane Nixon[2] J.E.Nixon@leeds.ac.uk

Moninder S Bhogal[5] M.S.Bhogal@leeds.ac.uk

Catherine Reynolds[2] C.Reynolds@leeds.ac.uk

Sarah Brown[2] Medsbro@leeds.ac.uk

On behalf of the CODIFI collaborators

The CODIFI collaborators are EA Nelson, J Nixon, S Brown, J Gray, J Firth, C Dowson, E Jude, T Dickie, C Amery, G Sykes, P Vowden, M Edmonds.

Affiliations

[1] School of Healthcare, University of Leeds, Leeds

[2] Clinical Trials Research Unit, University of Leeds, Leeds

[3] Leeds Institute of Rheumatic and Musculoskeletal Medicine, University of Leeds, Leeds

[4] Division of Medical Sciences, University of Oxford, Oxford

[5] School of Biomedical Sciences, University of Leeds, Leeds

Corresponding author:

E. Andrea Nelson, School of Healthcare

University of Leeds, LS2 9JT

E.A.Nelson@leeds.ac.uk

For peer review only

Abstract

Objective: To determine the extent of agreement and patterns of disagreement between wound swab and tissue samples in patients with an infected diabetic foot ulcer (DFU).

Design: Multi-centre, prospective, cross-sectional study.

Setting: Primary and secondary care foot ulcer/diabetic outpatient clinics and hospital wards across England.

Participants: Inclusion criteria: Consenting patients aged ≥18 years; diabetes mellitus; suspected infected DFU

Exclusion criteria: Clinically inappropriate to take either sample.

Interventions: Wound swab obtained using Levine's technique; tissue samples collected using a sterile dermal curette or scalpel.

Outcome measures:
Co-primary: Reported presence, and number, of pathogens per sample; prevalence of resistance to antimicrobials among likely pathogens
Secondary: Recommended change in antibiotic therapy based on blinded clinical review; adverse events; and, sampling costs.
Results: 400 consenting patients (79% male) from 25 centres.
Most prevalent reported pathogens were *Staphylococcus aureus* (43.8%), *Streptococcus* (16.7%), and other aerobic gram-positive cocci (70.6%). At least one potential pathogen was reported from 70.1% of wound swab and 86.1% of tissue samples. Pathogen results differed between sampling method in 58% of patients, with more pathogens and fewer contaminants reported from tissues.

The majority of pathogens were reported significantly more frequently in tissue than wound swab samples (p<0.01); with equal disagreement for *Staphylococcus aureus* and *Pseudomonas aeruginosa.* Blinded clinicians more often recommended a change in antibiotic regimen based on tissue compared with wound swab results (increase of 8.9%, 95% CI:2.65,15.3%). Ulcer pain and bleeding occurred more often after tissue collection versus wound swabs (pain: 9.3%,1.3%; bleeding: 6.8%,1.5%, respectively).

Conclusion: Reports of tissue samples more frequently identified pathogens, and less frequently identified non-pathogens compared with wound swab samples. Blinded clinicians more often recommended changes in antibiotic therapy based on tissue compared with wound swab specimens. Further research is needed to determine the effect of the additional information provided by tissue samples.

Study Registration: NRES Ref:11/YH/0078;UKCRN ID:10440;ISRCTN:52608451

For peer review only

For peer review only - http://bmjopen.bmj.com/site/about/guidelines.xhtml

Strengths and limitations of this study

- The first appropriately powered prospective study to assess agreement between these two methods of wound culture sampling.
- Investigates the relationship between baseline characteristics and agreement between the types of specimen using multivariable modelling.
- Included a sub-study to investigate the potential clinical relevance of the different amount of information gleaned from tissue and wound swab results by seeking opinion of blinded clinicians on whether the microbiology results indicate a need to change antibiotic therapy.
- This pragmatic study defined pathogens based on those reported by the clinical microbiology laboratory, so may not reflect all organisms/isolates identified.
- Tissue collection and sample culturing methods were not standardised across hospital laboratories.

Introduction

Diabetes mellitus is now a worldwide pandemic, with the prevalence in the United States now exceeding 14%[1]. In persons with diabetes, foot complications, most commonly ulceration related to peripheral sensory and motor neuropathy and peripheral arterial disease [2,3], occurs in 15% to 25% during their lifetime[4,5]. At presentation, over half of diabetic foot ulcers (DFU) are clinically infected [6] and foot infection precedes approximately 80% of non-traumatic lower limb amputations [4,7,8].

Infection is a clinical diagnosis made using classification guidelines to help clinicians to determine infection severity.[9] Antibiotics are commonly initiated immediately (empiric treatment) and the results of samples collected for identification of wound pathogens and their sensitivities are then used to tailor the antibiotic regimen, avoiding unnecessarily broad-spectrum therapy and antibiotic resistance.[10-12] Accurate culture results depend on collecting samples of infected tissue that is less likely to be contaminated by colonising flora. Sterile swabs for culture are widely available, quick and easy to use, and can be collected by most types of healthcare personnel. Unfortunately, wound swabs typically sample superficial flora, including colonizers or contaminants, and because of their construction (usually cotton wool) may fail to grow anaerobic or fastidious pathogens. Recognizing these limitations, many clinical microbiology laboratories

For peer review only

offer only minimal processing of wound swabs. Alternatively, specimens may be collected by obtaining tissue from the base of the wound; this requires slightly more skill and time, but may reveal more pathogens and be less susceptible to contamination with non-pathogens. Despite exhortations to obtain tissue rather than wound swab samples from most authoritative guidelines[9, 13, 14], many clinicians default to the wound swab method. Our previous systematic review identified few studies comparing results of wound swabs and tissue samples[15], and these had limitations including retrospective designs, inclusion of patients with various types of wounds, small cohorts and lack of contemporaneous sampling. Uncertainty has not been resolved in subsequent studies. One[16] retrospectively reviewed 54 pairs of samples (from people with DFU but not all of whom had a wound infection) and reported that wound swabs detected more species than tissue samples -- finding additional species in 11.2% of cases, fewer species in 9.0% of cases, and completely different organisms in 6.7%. In a second study 50 patients with an infected DFU had both swab and tissue samples taken; with the latter considered the 'gold-standard,' wound swabs had 100% sensitivity but <20% specificity[17]. A third study, which collected specimens from 56 patients with an infected DFU, noted that wound swabs missed organisms identified from tissue specimens, especially gram-negative bacteria, in patients with more severe infections[18].

A further limitation of the published literature is that investigators have made the assumption that tissue specimens are the 'gold-standard,' for sampling, but this method may also miss wound flora. Hence, we proposed a study to assess agreement and extent of disagreement between the two methods of collecting wound specimens, by comparing the pathogens isolated from each method from the same wound.

Study Design

We assessed the agreement between culture results of tissue and wound swab samples in patients with a suspected infected DFU. We have published a detailed description of the study methods.[19]

For peer review only - http://bmjopen.bmj.com/site/about/guidelines.xhtml

This was a multicentre, cross-sectional study of 400 people with diabetes mellitus (79% were male) in English primary and secondary care foot ulcer/diabetic outpatient clinics and hospital wards. Foot ulcer infection was diagnosed clinically based on signs and symptoms using Infectious Diseases Society of America /International Working Group on the Diabetic Foot (IDSA/IWGDF) criteria; patients were eligible for enrolment if the clinician evaluating them planned to treat them with antibiotic therapy. Consenting patients had a wound swab and tissue sample taken from the same foot ulcer. These were processed and reported by the usual local clinical microbiology laboratory so that the information gathered would be relevant for clinical practice.

Co-primary endpoints were the extent of agreement between wound swab and tissue sampling for three microbiological parameters: 1) presence of isolates likely to be pathogens; 2) the number of bacterial pathogens reported per sample; and, 3) the prevalence among likely pathogens of resistance to antimicrobials.

In addition we investigated the clinical usefulness of the information provided by tissue versus wound swab samples, using a blinded clinical review panel to interpret the microbiology results. Secondary objectives considered sampling-related adverse effects and the costs of sampling.

In a separate sub-study, we investigated the clinical outcomes at 12 months post sampling and explored the prognostic factors related to ulcer healing.[20]

Eligibility Criteria

Patients were eligible if they had: a diagnosis of diabetes mellitus (type 1 or 2); were at least 18 years old; and, had a suspected infected DFU (with or without bone infection, based on clinical signs and symptoms using Infectious Diseases Society of America / International Working Group on the Diabetic Foot (IDSA / IWGDF) criteria and the judgement of the investigator). Patients were excluded if: the treating clinician deemed it inappropriate to take a tissue or wound swab sample for any reason; the patient had previously been recruited into the study; or, they were unwilling or unable to provide informed consent. Patients were not excluded if they were currently being, or had recently been, treated with antimicrobial therapy.

Assessments

For peer review only

Sample Acquisition

We trained clinicians at all centres to collect samples using the UK Health Protection Agency (HPA) standards [21,22] which were subsequently updated [23, 24], via site visits and an e-Learning package that we developed for this purpose.[25] After wound cleansing and debridement (if required), a physician, nurse or podiatrist first obtained the wound swab sample from the infected ulcer using Levine's technique.[26] A tissue sample was subsequently collected using a sterile dermal curette or scalpel and placed in the transport medium used locally. All samples were transferred to, and processed by, the centre's local clinical microbiology laboratory. [21-24] Study samples received no special labelling or processing.

Clinical Assessments

Baseline data included a medical history and examination, including for any signs or symptoms of wound infection, previous treatments, and classifying the current status of the foot ulcer using the PEDIS,[27] Wagner,[28] and Clinical Signs and Symptoms Classification of Infection systems [29], and level of pain in the ulcer immediately after each sample was obtained. Investigators reported adverse events associated with sample collection.

Centre differences questionnaire

Each participating site, including its microbiology laboratory, completed a questionnaire regarding how they: acquired samples for culture; transported them to the laboratory; analysed the specimens; and, reported the results to clinicians. We also requested that they report their local antibiotic protocols, to allow evaluation of any potential differences among centres.

Clinical Panel Review

We compared the proportion of patients for whom the antibiotic regimen actually prescribed by the attending medical team was 'appropriate,' based on culture and sensitivity results of wound swab or tissue samples. We sent microbiology results, along with a record of the empirical antimicrobial regimen prescribed, for the first 250 recruited patients (three were subsequently excluded due to protocol deviation or incomplete review) to a panel of 13 senior clinicians who worked with a diabetic foot team and had antibiotic prescribing privileges. Each clinician received the results of cultures of patients wound swab or tissue sample on different occasions, and were

For peer review only - http://bmjopen.bmj.com/site/about/guidelines.xhtml

blinded to whether results were from a tissue or wound swab specimen, and if they were from the same or different patients. Clinicians were asked:

1. ‘Are there any pathogens identified in the lab report that are not covered by the prescribed antimicrobial regimen? (Yes/No)’
2. ‘If you answered ‘yes’ to question 1, would knowing this information lead you to prescribe an alternative antibiotic regimen for this patient? (Yes/No)’.

For peer review only

Sample Size

Our sample size was based on the primary outcome of the reported ‘presence or absence of a pathogen’. Our target sample size was 400, as we calculated that 399 patients would provide 80% power to detect a difference of ≥3% in the reported presence of a given pathogen, if overall prevalence was 10%, with 5% disagreement between the wound swab and tissue samples, using a two-sided McNemar’s test at the 5% level of significance. This level of agreement would also provide a kappa statistic of 0.7. This calculation is based on lower prevalence organisms, such as *Pseudomonas aeruginosa*[30], hence the power was higher for more prevalent species.

STATISTICAL ANALYSIS

All tests of statistical significance were two-sided and based on results from the evaluable population, with p-values and 95% confidence intervals (CI) provided as appropriate.

The various microbiology laboratories reported pathogens at a range of taxonomic levels, which we grouped by a previously developed scheme designed to report statistics meaningfully, i.e. by genus, species, etc. For pathogens with a prevalence >8% we generated cross-tabulations of reported presence in wound swab and tissue: overall percentage prevalence; agreement and disagreement; unadjusted kappa for agreement; prevalence and bias adjusted kappa (PABAK) for agreement; prevalence difference (tissue – wound swab, and 95% CI); and McNemar's test for differences. As the participating laboratories used a number of scales to quantify the extent of growth of a pathogen (for example, +/++/+++; +/++/+++/++++; scanty/light/moderate/heavy; scanty/+/++/+++; light, moderate, heavy), we derived these onto one three-point scale reported

For peer review only - http://bmjopen.bmj.com/site/about/guidelines.xhtml

as +/++/+++. We used the derived data to tabulate the extent of bacterial growth (none, + to +++) and calculate weighted kappa statistics.

We pre-specified baseline factors to investigate their relevance in determining agreement between sample results, including: type of ulcer (ischemic or neuro-ischaemic versus neuropathic); Wagner grade of ulcer (1-5); recent antimicrobial therapy; and wound duration. We generated an overall summary of pathogens[31], and used univariable multinomial regression by centre to determine whether agreement was influenced by any of these factors.

Using univariable ordinal regression modelling we assessed the influence of baseline factors on the number of pathogens as follows: tissue sampling (compared to wound swab) had 2 or more extra pathogens reported; tissue sampling had 1 extra pathogen reported; tissue and wound swab sampling had the same number of pathogens reported; wound swab sampling had 1 or more extra pathogens reported. In both regression analyses, we included centre as a random effect and multiple imputation to impute missing baseline factors.

For the clinical panel study of appropriateness of antibiotic treatment we summarised whether the pathogens reported were, or were not, covered by the actual treating clinician's prescribed antimicrobial regimen. We also asked if, in the blinded clinician's opinion, a change in antibiotic therapy was required. We used McNemar's test to identify whether one sampling method identified more patients requiring a change in therapy than the other.

RESULTS

Recruitment

Between 15th November 2011 and 15th May 2013 we screened 680 patients, and enrolled 401 patients from 25 centres. We excluded one patient whose consent was lost and 5 for whom one or more sample was lost or misused, resulting in a full analysis set of 400 patients and an evaluable population of 395 patients (Figure 1).

Demographics

For peer review only - http://bmjopen.bmj.com/site/about/guidelines.xhtml

The recorded demographic characteristics of patients screened and those ultimately recruited were comparable. Most patients were recruited from outpatient clinics (79.8%) and were male (79.0%). Recruited patients had a median age of 63 years (range 26 – 99), a median duration of diabetes of 16.8 years (IQR 9-23), and median duration of their index ulcer of 5.6 months (IQR 0.7 – 6.0). Before sampling, 60.3% had an antimicrobial dressing or agent applied on the suspected infected ulcer, and 46.8% had received some type of systemic antibiotic therapy. After enrolment, 93.5% of patients received systemic antibiotic therapy (Table 1).

Microbiology results

Culture results yielded 79 different types of microbial isolates. Among the wound swab samples, there were no isolates reported from 20.0% and non-pathogenic isolates from 9.9%. Among tissue samples, there were no isolates reported in 10.1% and non-pathogenic isolates from 3.8%. (Table 2)

The most frequently reported groups of pathogens were: gram-positive cocci (70.6%); gram-negative bacilli (36.7%); *Enterobacteriaceae*, including coliforms (26.6%); obligate anaerobes (23.8%); and, gram-positive bacilli (11.1%). The most frequently reported pathogens were: *Staphylococcus aureus* (43.8%, of which 8.1% were methicillin-resistant); *Streptococcus* (16.7%); *Enterococcus* (14.9%); coagulase-negative *Staphylococcus* (12.2%); *Corynebacterium* (9.4%); and, *Pseudomonas aeruginosa* (8.6%). All other genus and species level pathogens had a combined prevalence <6%. (Table 2)

Primary Endpoints

Summary of pathogens reported

For 58.0% of patients there was a difference in the pathogens reported by the two sampling techniques. The wound swab reported additional pathogens to those in the tissue sample in 8.1%; the tissue sample reported additional pathogens to those in the wound swab in 36.7%; and, the tissue and wound swab samples reported different pathogens, with or without overlap, in 13.2%.

For peer review only - http://bmjopen.bmj.com/site/about/guidelines.xhtml

For peer review only

Reported presence of pathogens

The majority of pathogens were reported significantly more frequently in the tissue than the wound swab samples ($p<0.01$). For isolates of *S. aureus* and *Pseudomonas aeruginosa,* however, there was equal disagreement, meaning that for the same number of patients wound swabbing missed a pathogen reported by tissue sampling, as there were pathogens missed by tissue sampling but reported by wound swabbing. A full cross-tabulation of the reported presence of all of these pathogens is shown in Table 2, with statistical analyses presented in Table 3.

We examined whether the outcome "both wound swab and tissue report the same pathogens" was related to any of several potentially important patient baseline variables (Table 4). Based on a summary of our results we performed a univariable multinomial analysis and found that none of the baseline factors examined had a significant effect on overall agreement.

Reported presence of antimicrobial resistance among likely pathogens

We investigated the reported presence of three common antimicrobial-resistant pathogens using two sampling methods. Methicillin-resistant *S. aureus* (MRSA) was reported in 6.8% of wound swabs and 7.8% of tissue samples, a difference of 1.0% (95% CI: -0.2-2.8%, McNemar's exact p-value=0.219). Vancomycin-resistant *Enterococcus* were reported in only 1 (0.3%) patient (detected by both wound swab and tissue). No methicillin-resistant coagulase-negative staphylococci was reported.

Number of pathogens reported per sample

Comparing the number of pathogens isolated from tissue versus wound swab specimens, both had a median 1.0 pathogen per sample, but the means were 1.5 and 1.0 and the maximum numbers were 6 and 4 pathogens, respectively. A greater proportion of wound swab samples reported no pathogens compared to tissue samples (29.9% vs. 13.9% respectively). In terms of number of pathogens reported for the tissue versus the wound swab sample, for 49.6% of patients they were the same, for 41.5% there was at least one more pathogen was reported from the tissue than the wound swab sample, and for 8.9% of there was at least one more pathogen reported from the wound swab than the tissue sample.

For peer review only - http://bmjopen.bmj.com/site/about/guidelines.xhtml

By univariable ordinal analysis we found that patients' tissue samples were reported to have 2 or more additional pathogens significantly more often if their ulcer was present for ≥56 days than if it was present <56 days (OR 1.56, 95%CI: 1.05-2.33, p=0.024).

Clinical Panel Review

In 73.3% of the cases reviewed by the blinded panel there was moderate agreement on the requirement for a change in therapy between the wound swab and the tissue samples (kappa 0.45, 95%CI: 0.34-0.56). In 17.8% of cases the blinded clinician indicated that the tissue sample results would lead to a recommendation of change in therapy, while the wound swab sample would not indicate a need for change. In 8.9% of cases the blinded clinician indicated that the wound swab result would lead to a change in therapy whereas the tissue sample would not (increase of 8.9%, 95%CI: 2.7-15.3%).

Adverse Events

Investigators reported "bleeding of concern" during sample collection in 30 (7.6%) of the recruited patients; it was attributed to the wound swab in 6 patient (1.5%) and to tissue sampling in 27 patients (6.8%). Higher levels of pain after either wound swab or tissue sampling was reported by 42 (10.5%) of patients. Of these 5 (1.3%) patients reported worse pain after wound swabbing compared to tissue sampling, and 37 (9.3%) patients reported worse pain after tissue sampling compared to wound swabbing.

Centre differences

We received responses to our questionnaires from 22 centres. Regarding the tissue sampling technique, one site used a dermal curette to collect tissue samples and others used a scalpel. There were no differences in the amount of time for a wound swab and a tissue sample to reach the microbiology laboratory from the clinic and no difference in the time it took from the receipt of the samples to processing. Among responding centres, 4 of 17 (23.5%) reported slightly more urgent processing of tissue samples.

For peer review only

Microbiology laboratories performed a Gram-stained smear of the specimen more frequently for tissue than wound swab samples; of 19 laboratories, 9 (47.4%) did this for tissue only, 3 (15.8%) did it for both samples, and 6 (31.6%) did not routinely perform Gram-staining (but offered it on request in 1 laboratory). Of 18 laboratories, 10 (55.6%) reported all isolates grown from a tissue sample but tailored wound swab sample reports according to clinical details and likely microbiological significance of the isolates. Centre differences were apparent in the multinomial and ordinal regression analysis where its inclusion improved the fit of both models ($p<0.001$).

Because only two microbiology laboratories provided data on the cost of processing specimens, it was not possible for us to do an analysis by specimen type.

Discussion

To our knowledge this is the largest comparison of the two main methods of sampling an infected diabetic foot ulcer, the first to report detailed data on paired samples for each pathogen from paired samples and the first to examine the relationship between baseline characteristics and agreement between microbiology results by types of specimen using multivariable modelling.

We found that tissue sampling had a higher yield than wound swab specimens, hence providing more information on wound flora. While tissue sampling overall detected more organisms than wound swabs, both techniques missed some organisms. Thus, to some degree they provide complementary information and both techniques may be useful. The differences in the results of the two sampling techniques may be related to: the tissue specimen providing a greater yield of organisms at collection; a lower rate of bacterial isolates dying during specimen transport; or, differences in the way the microbiology laboratory handled or reported the culture results. In settings where obtaining specimens by wound swab remains the standard method, until we determine the clinical impact of choosing tissue over swab sampling, we suggest examining methods to increase the yield from wound cultures.

For peer review only - http://bmjopen.bmj.com/site/about/guidelines.xhtml

For chronic wounds, there is no gold standard method of diagnosing infection. The minority of samples in our study that reported no pathogens may reflect either a false positive diagnosis of infection[32] or a false negative culture related to the use of antimicrobial dressings and antibiotics prior to sampling. Alternatively, this finding may be related to: improper sampling technique (e.g., not sufficiently expressing tissue fluid in Levine's technique[26]); transport media that fail to maintain the viability of wound swab pathogens; or, a decision by the microbiology laboratory to report only pathogens that they deemed clinically significant.

A key clinical issue is how much, and what type of, information on ulcer flora is useful for clinicians managing patients with an infected diabetic foot ulcer. While clinicians want to optimally target their antibiotic therapy, providing microbiology reports listing many organisms, including likely non-pathogenic or unusual isolates present in low numbers, may confuse rather than aid decision-making. We do not know, based on our results or the available literature, if antibiotic treatment based upon a more detailed microbiogram helps select an antimicrobial regimen that increases the likelihood of, or time to, resolution of infection, or the prevention of treatment associated antibiotic resistance.

We found that when blinded clinicians were presented with tissue, as opposed to wound swab microbiology reports they were more likely to recommend a change in antibiotic therapy. This suggests that the additional information tissue specimens provide could lead to more tailored antimicrobial regimens. We do not know, however, if this theoretical finding would be confirmed in clinical practice.

It is certainly important to adequately cover all likely pathogens in a potentially limb-threatening problem like diabetic foot infection. However, given the global emergency associated with antibiotic-resistance related to over-use of this precious resource, we are cautious about recommending a wholesale change to adoption of tissue sampling as theoretically this is a technique that may lead to unnecessarily broad-spectrum prescribing. Furthermore, the bacterial flora in the wound at the time of sampling may differ from those present days later after empiric antibiotic therapy, when culture results are reported, potentially reducing the utility of this information.

For peer review only

This study has several strengths. We provided all centres with training on appropriate techniques for wound swab and tissue sampling in an effort to minimise between sample, and between centre, differences. We prospectively enrolled a large number of patients at many clinical sites, using a carefully-defined protocol that required obtaining contemporaneous dual specimens on each patient. The study also has high external validity, as we had minimal exclusion criteria, we recruited patients in usual practice settings, members of the attending clinical teams obtained the samples, and the local laboratories processed the specimens.

There were, of course, some potential weaknesses of the study. There were differences among laboratories in tissue collection and sample culturing methods. These differences reflect the pragmatic nature of the study and ensures the results are generalisable to NHS centres and laboratories across England. Furthermore, only a small minority of patients (7%) were recruited from primary care (as opposed to specialty clinic or inpatient) centres. This limited our ability to investigate whether there was any difference in the extent of agreement in the reporting of pathogens between primary and secondary care sites.

Previous reports comparing wound swab to tissue specimens have been small, single-centre studies, and produced mixed results. One retrospective study of 89 concomitantly obtained pairs of samples from 54 patients with diabetic foot ulcers (87% clinically infected), [16] found that culture results of superficial wound swabs did not correlate well with those obtained from deep tissue, but they summarised their results in terms of predictive value for infection, for which there is no good evidence (deep tissue samples are an imperfect gold standard for diagnosing infection). Another study of 50 patients with an infected diabetic foot ulcer [17] that compared culture results of tissue against wound swab specimens found that reports agreed in only 50% of patients. In another study of 56 patients with diabetic foot infection, grouped according to the PEDIS grading system, [18] wound swab culturing identified all micro-organisms isolated from the corresponding deep tissue culture in 90% of grade 2 wounds, and in 41.4% and 41.2% for grade 3 and 4 wounds respectively.

We believe our results demonstrate the increased yield from tissue compared to wound swab specimens; the maximum information would be available when reports from both samples are obtained. Combined with the currently available literature, this reinforces the recommendations

For peer review only - http://bmjopen.bmj.com/site/about/guidelines.xhtml

that tissue samples are preferred over swab specimens if one method is to be selected. However, current guidelines don't recognise the complementarity of information when both methods are used. What is still needed is further research on whether this increased information from tissue sampling results in more appropriate prescribing or better resolution of infection or improved wound healing. Furthermore, we need more research on whether molecular approaches that provide extended views of the microbiome in conjunction with new developments in near patient testing improve clinical outcomes and antibiotic stewardship. Results of these further studies would inform the most appropriate method of obtaining specimens from diabetic foot ulcers.

END

For peer review only

For peer review only - http://bmjopen.bmj.com/site/about/guidelines.xhtml

Funding: This work was supported by the National Institute for Health Research (NIHR) Health Technology Assessment (HTA) programme (project number 09/75/01).

Disclaimer: The views and opinions expressed herein are those of the authors and do not necessarily reflect those of the HTA programme, NIHR, NHS or the Department of Health.

Competing interests: All authors declare they have no competing interests

Ethics Approval: CODIFI received ethical approval from the Sheffield NRES Committee (Ref: 11/YH/0078) and each enrolling site obtained local ethical approval prior to commencing recruitment.

Data Sharing statement: Requests for data should be made to the corresponding author

Authors' Contributions: Professor E. Andrea Nelson (Professor of Wound Healing) was the chief investigator for the study and is the guarantor. She initiated the study, led the grant application development and the study team, and was involved in drafting and revising this paper critically for intellectual content. Miss Alexandra Wright-Hughes (Senior Statistician) was a member of the study team, contributed to the drafting of the statistical analysis plan, undertook the statistical analyses, drafted the statistical results and contributed to the drafting and revising paper for intellectual content. Dr Michael Backhouse (NIHR Research Fellow, Podiatrist) was the first clinical study coordinator, and a member of the study team, initiated sites for recruitment, contributed to the drafting of the study report and revised it for intellectual content. Professor Benjamin A. Lipsky (Professor of Medicine) was a co-applicant on the study grant, a member of the study team, contributed to the drafting of this paper and revised it for intellectual content. Professor Jane Nixon (Professor of tissue viability) was a co-applicant on the study grant, was a member of the study team, the lead for the Clinical Trials Unit activity and responsible for these aspects, she contributed to the drafting of the manuscript and reviewed it for intellectual content. Dr Moninder Bhogal (Senior Trial Coordinator) was the trials unit study coordinator, and a member of the study team, he was responsible for ethics and governance applications and coordinated the study team, he contributed to the drafting of the study report and revised it for intellectual content. Miss Catherine Reynolds (Senior Data Manager), was the data manager and a member of the study team, responsible for data quality, contributed to the

For peer review only

For peer review only - http://bmjopen.bmj.com/site/about/guidelines.xhtml

drafting of the study report and revised it for intellectual content. Miss Sarah Brown (Principal Statistician) the supervising statistician, was a co-applicant on the study grant, and a member of the study team, contributed to the drafting of the statistical analysis plan, oversaw the statistical analyses and preparation of the statistical results, she contributed to the drafting of the manuscript and reviewed it for intellectual content.

For peer review only

Acknowledgements

We thank members of the study steering committee (Prof J Deeks, Prof R Cooper, Prof R Gadsby, Prof AM Keenan, Mrs C Thomas).

The CODIFI collaborators were EA Nelson, J Nixon, S Brown, J Gray, J Firth, C Dowson, E Jude, T Dickie, C Amery, G Sykes, P Vowden, M Edmonds

For peer review only

References

1. Menke A CS, Geiss L, Cowie CC. Prevalence of and Trends in Diabetes Among Adults in the United States, 1988-2012. JAMA 2015; 314(10): 1021-9.

2. Vinik AI, Maser RE, Mitchell BD, Freeman R. Diabetic autonomic neuropathy. Diabetes Care 2003; 26(5): 1553-1579.

3. Richard JL, Lavigne JP, Sotto A. Diabetes and foot infection: more than double trouble. Diabetes/Metabolism Research and Reviews 2012; 28: 46-53.

4. Singh N, Armstrong DG, Lipsky BA. Preventing foot ulcers in patients with diabetes. JAMA 2005; 293(2): 217-228.

5. Lavery L, Armstrong D, Wunderlich R, Tredwell J, Boulton A. Diabetic foot syndrome: evaluating the prevalence and incidence of foot pathology in Mexican Americans and non-Hispanic whites from a diabetes disease management cohort. Diabetes Care 2003; 26(5): 1435-1438.

6. Prompers L, Huijberts M, Schaper N, et al. Resource utilisation and costs associated with the treatment of diabetic foot ulcers. Prospective data from the Eurodiale Study. Diabetologia 2008; 51(10): 1826-34.

7. Reiber G. The epidemiology of diabetic foot problems. Diabetic Medicine 1996; 13: S6.

8. Armstrong DG, Lavery LA, Quebedeaux TL, Walker SC. Surgical morbidity and the risk of amputation due to infected puncture wounds in diabetic versus nondiabetic adults. Journal of the American Podiatric Medical Association 1997; 87(7): 321-6.

9. Lipsky BA, Aragon-Sanchez J, Diggle M, Embil J, Kono S, Lavery L, Senneville E, Urbancic-Rovan V, Van Asten S, Peters EJG, on behalf of the International Working Group on the Diabetic Foot (IWGDF) IWGDF guidance on the diagnosis and management of foot infections in persons with diabetes. Diabetes Metab Res Rev 2016; 32(Suppl. 1): 45–74

10. Lipsky B. Empirical therapy for diabetic foot infections: are there clinical clues to guide antibiotic selection? Clinical Microbiology and Infection 2007; 13(4): 351-3.

11. Lipsky BA. A report from the international consensus on diagnosing and treating the infected diabetic foot. Diabetes/Metabolism Research and Reviews 2004; 20(S1): S68-S77.

12. Lipsky BA, Berendt AR, Deery HG, et al. Diagnosis and treatment of diabetic foot infections. Clinical Infectious Diseases 2004; 39(7): 885-910.

13. Lipsky BA, Berendt AR, Cornia PB, et al. 2012 Infectious Diseases Society of America clinical practice guideline for the diagnosis and treatment of diabetic foot infections. Clinical Infectious Diseases 2012; 54(12): e132-73.

14. NICE. Diabetic foot problems: prevention and management. http://www.nice.org.uk/NG19

15. Nelson EA, O'Meara S, Craig D, et al. A series of systematic reviews to inform a decision analysis for sampling and treating infected diabetic foot ulcers. Health Technology Assessment 2006; 10(12): iii-iv, ix-x, 1-221.

For peer review only

16. Mutluoglu M, Uzun G, Turhan V, Gorenek L, Ay H, Lipsky BA. How reliable are cultures of specimens from superficial swabs compared with those of deep tissue in patients with diabetic foot ulcers? Journal of Diabetes and its Complications 2012; 26(3): 225-9.

17. Demetriou M, Papanas N, Panopoulou M, Papatheodorou K, Bounovas A, Maltezos E. Tissue and swab culture in diabetic foot infections: neuropathic versus neuroischemic ulcers. The international journal of lower extremity wounds 2013; 12(2): 87-93.

18. Huang Y CY, Zou M, Luo X, Jiang Y, Xue Y, Gao F. A Comparison of Tissue versus Swab Culturing of Infected Diabetic Foot Wounds. Int J Endocrinol 2016; 8198714.

19. Nelson EA, Backhouse MR, Bhogal MS, et al. Concordance in diabetic foot ulcer infection. BMJ Open 2013; 3(1).

20. Ndosi M, Wright-Hughes A, Brown S, Backhouse M, Lipsky BA, Bhogal M, Reynolds C, Vowden P, Jude E, Nixon J, Nelson EA. Prognosis of the infected diabetic foot ulcer: a 12-month prospective observational study. Diabetic Medicine. 2017 Aug 25.21. Health Protection Agency (2009). Investigation of skin, superficial and non-surgical wound swabs. National Standard Method BSOP 11 Issue 5.
http://www.hpa-standardmethods.org.uk/pdf_sops.asp

22. Health Protection Agency (2009). Investigation of tissues and biopsies. National Standard Method. http://www.hpa-standardmethods.org.uk/pdf_sops.asp

.

23. *Public Health England. Investigation of skin, superficial and non-surgical wound swabs.* Standards for microbiology investigations *2016; SMI B11 Issue 6.1.*
https://www.gov.uk/government/collections/standards-for-microbiology-investigations-smi

24. *Public Health England.* Investigation of tissues and biopsies from deep seated sites and organs. Standards for microbiology investigations 2017; *SMI B17 Issue 6.2.*
https://www.gov.uk/government/collections/standards-for-microbiology-investigations-smi

25. Randell R, Backhouse MR, Nelson EA. Videoconferencing for site initiations in clinical studies: Mixed methods evaluation of usability, acceptability, and impact on recruitment. Informatics for Health and Social Care 2015: 1-11.

26. Levine NS, Lindberg RB, Mason AD, Jr., Pruitt BA, Jr. The quantitative swab culture and smear: A quick, simple method for determining the number of viable aerobic bacteria on open wounds. The Journal of Trauma 1976; 16(2): 89-94.

27. Schaper N. Diabetic foot ulcer classification system for research purposes: a progress report on criteria for including patients in research studies. Diabetes/Metabolism Research and Reviews 2004; 20(S1): S90-S5.

28. Wagner Jr F. The diabetic foot. Orthopedics 1987; 10(1): 163-72.

29. Gardner S, Frantz R, Troia C, et al. A tool to assess clinical signs and symptoms of localized infection in chronic wounds: development and reliability. Ostomy/Wound Management 2001; 47(1): 40.

For peer review only

30. Pellizzer G, Strazzabosco M, Presi S, et al. Deep tissue biopsy vs. superficial swab culture monitoring in the microbiological assessment of limb-threatening diabetic foot infection. Diabetic Medicine 2001; 18(10): 822-7.

31. Slater R, Lazarovitch T, Boldur I, et al. Swab cultures accurately identify bacterial pathogens in diabetic foot wounds not involving bone. Diabetic Medicine 2004; 21(7): 705-9.

32. Edmonds M, Foster A. The use of antibiotics in the diabetic foot. Am J Surg 2004; 187(5A): 25s-8s.

Table 1 Baseline characteristics of enrolled patients

Characteristic	Clinical Values	Full Analysis Set (n=400)
**Age (years)**	Mean (SD)	63·1 (13·3)
	Median, range and (IQR)	63·0 [26-99] (54·0, 73·0)
**Sex**	Male	316 (79·0%)
	Female	84 (21·0%)
**Ethnicity**	White	377 (94·3%)
	Other	23 (5·7%)
**Site of recruitment**	Hospital ward	53 (13·3%)
	Outpatient clinic	319 (79·8%)
	Community clinic	28 (7·0%)
**Diabetes type**	Type 1	58 (14·5%)
	Type 2	342 (85·5%)
**Duration of diabetes (years)**	N Missing	3
	Mean (SD)	16·8 (11·0)
	Median, range and (IQR)	15·0 [0·04-57] (9·0, 23·0)
**Diabetes treatment details**	Oral hypoglycaemic agent	107 (27·8%)
	Insulin	168 (43·6%)
	Oral hypoglycaemic agent & Insulin	109 (28·3%)
	Other	1 (0·3%)
	None	15 (3·8%)
**Number of foot ulcers**	1	268 (67·0%)
	≥2	132 (33·0%)
**Duration of index ulcer (months)**	N Missing	4
	Mean (SD)	5·58 (12·28)
	Median, [range] and (IQR)	1·84 [0·1-144·0] (0·69, 6·00)
**Aetiology of index ulcer**	Ischemic	14 (3·5%)
	Neuropathic	202 (50·5%)
	Ischemic & Neuropathic	182 (45·5%)
	Missing	2 (0·5%)
**Antimicrobial dressing on ulcer**	Yes	241 (60·3%)
	No	154 (38·5%)
	Missing	5 (1·3%)
**Patient already on systemic antibiotics**	Yes	187 (46·8%)
	No	194 (48·5%)
	Missing	19 (4·8%)
**Patient on antibiotics immediately post sampling**	Yes	374 (93·5%)
	No	26 (6·5%)
**Grade (Wagner scale)[a]**	Grade 1	136 (34·0%)
	Grade 2	134 (33·5%)
	Grade 3	122 (30·5%)
	Grade 4	7 (1·8%)
	Grade 5	1 (0·3%)

[a] Grade 1 - Superficial diabetic ulcer (partial or full thickness); Grade 2 – Ulcer extension ligament, tendon, joint capsule, or deep fascia without abscess or osteomyelitis; Grade 3 – Deep ulcer with abscess, osteomyelitis or joint sepsis; Grade 4 – Gangrene localized to portion of forefoot or heel; Grade 5 – Extensive gangrenous involvement of the entire foot·

For peer review only - http://bmjopen.bmj.com/site/about/guidelines.xhtml

Table 2 Cross tabulation of reported presence of at least one pathogen and pathogens with >8% prevalence in order of taxonomic rank and prevalence

**Pathogen (Overall prevalence)**			**Tissue Results**	**Tissue Results**	
			Not reported	***Reported***	***Total***
**At least one pathogen (88·1%)**	***Swab***	***Not reported***	47 (11·9%)	71 (18·0%)	118 (29·9%)
	Swab	***Reported***	8 (2·0%)	269 (68·1%)	277 (70·1%)
		Total	55 (13·9%)	340 (86·1%)	395 (100·0%)
**Gram-positive cocci (70·6%)**	***Swab***	***Not reported***	116 (29·4%)	68 (17·2%)	184 (46·6%)
	Swab	***Reported***	14 (3·5%)	197 (49·9%)	211 (53·4%)
		Total	130 (32·9%)	265 (67·1%)	395 (100·0%)
**Gram-negative bacilli (36·7%)**	***Swab***	***Not reported***	250 (63·3%)	49 (12·4%)	299 (75·7%)
	Swab	***Reported***	12 (3·0%)	84 (21·3%)	96 (24·3%)
		Total	262 (63·3%)	133 (33·7%)	395 (100·0%)
**Enterobacteriacea (including coliforms) (26·6%)**	***Swab***	***Not reported***	290 (73·4%)	37 (9·4%)	327 (82·8%)
	Swab	***Reported***	14 (3·5%)	54 (13·7%)	68 (17·2%)
		Total	304 (77·0%)	91 (23·0%)	395 (100·0%)
**Obligate anaerobes (23·8%)**	***Swab***	***Not reported***	301 (76·2%)	46 (11·6%)	347 (87·8%)
	Swab	***Reported***	19 (4·8%)	29 (7·3%)	48 (12·2%)
		Total	320 (81·0%)	75 (19·0%)	395 (100·0%)
**Gram-positive bacilli (11·1%)**	***Swab***	***Not Reported***	351 (88·9%)	40 (10·1%)	391 (99·0%)
	Swab	***Present***	1 (0·3%)	3 (0·8%)	4 (1·0%)
		Total	352 (89·1%)	43 (10·9%)	395 (100·0%)
***Streptococcus* (16·7%)**	***Swab***	***Not reported***	329 (83·3%)	18 (4·6%)	347 (87·8%)
	Swab	***Reported***	5 (1·3%)	43 (10·9%)	48 (12·2%)
		Total	334 (84·6%)	61 (15·4%)	395 (100·0%)
***Enterococcus* (excluding VRE) (14·9%)**	***Swab***	***Not reported***	336 (85·1%)	34 (8·6%)	370 (93·7%)
	Swab	***Reported***	6 (1·5%)	19 (4·8%)	25 (6·3%)
		Total	342 (86·6%)	53 (13·4%)	395 (100·0%)
**Coagulase-negative *Staphylococcus* (12·2%)**	***Swab***	***Not reported***	347 (87·8%)	39 (9·9%)	386 (97·7%)
	Swab	***Reported***	1 (0·3%)	8 (2·0%)	9 (2·3%)
		Total	348 (88·1%)	47 (11·9%)	395 (100·0%)
***Corynebacterium* (9·4%)**	***Swab***	***Not reported***	358 (90·6%)	33 (8·4%)	391 (99·0%)
	Swab	***Reported***	1 (0·3%)	3 (0·8%)	4 (1·0%)
		Total	359 (90·9%)	36 (9·1%)	395 (100·0%)
***Pseudomonas aeruginosa*(8·6%)**	***Swab***	***Not reported***	361 (91·4%)	8 (2·0%)	369 (93·4%)
	Swab	***Reported***	8 (2·0%)	18 (4·6%)	26 (6·6%)
		Total	369 (93·4%)	26 (6·6%)	395 (100·0%)
***Staphylococcus aureus* (excluding MRSA) (35·7%)**	***Swab***	***Not reported***	254 (64·3%)	16 (4·1%)	270 (68·4%)
	Swab	***Reported***	16 (4·1%)	109 (27·6%)	125 (31·6%)
		Total	270 (68·4%)	125 (31·6%)	395 (100·0%)
**Methicillin-resistant *S· aureus* (8·1%)**	***Swab***	***Not reported***	363 (91·9%)	5 (1·3%)	368 (93·2%)
	Swab	***Reported***	1 (0·3%)	26 (6·6%)	27 (6·8%)
		Total	364 (92·2%)	31 (7·8%)	395 (100·0%)

Abbreviations: VRE= vancomycin-resistant enterococcus, MRSA= methicillin-resistant *Staphylococcus aureus*

For peer review only - http://bmjopen.bmj.com/site/about/guidelines.xhtml

For peer review only

Table 3 Summary of agreement and disagreement statistics for most prevalent pathogens and the report of at least one pathogen

	Overall prevalence	Overall disagreement	Difference (95% CI)*	McNemar's P Value	Overall agreement	Unadjusted Kappa (95% CI)	PABAK
**At least one pathogen**	88·1%	20·0%	15·9% (11·8%, 20·1%)	<0·0001	80·0%	0·44 (0·34, 0·53)	0·60
**Gram-positive cocci**	70·6%	20·8%	13·7% (9·4%, 18·0%)	<0·0001	79·2%	0·57 (0·50, 0·65)	0·58
**Gram-negative bacilli**	36·7%	15·4%	9·4% (5·6%, 13·1%)	<0·0001	84·6%	0·63 (0·55,0·71)	0·69
**Enterobactereacea (Including coliforms)**	26·6%	12·9%	5·8% (2·3%,9·3%)	0·0013	87·1%	0·60 (0·50,0·70)	0·74
**Obligate anaerobes**	23·8%	16·5%	6·8% (2·9%, 10·8%)	0·0008	83·5%	0·38 (0·26,0·50)	0·67
**Gram-positive bacilli**	11·1%	10·4%	9·9% (6·9%, 13·5%)	<0·0001**	89·6%	0·11 (-0·01,0·23)	0·79
Streptococcus	16·7%	5·8%	3·3% (0·9%, 5·6%)	0·0067	94·2%	0·76 (0·66,0·85)	0·88
***Enterococcus* (exc· VRE)**	14·9%	10·1%	7·1% (4·0%, 10·1%)	<0·0001	89·9%	0·44 (0·30,0·58)	0·80
**Coagulase-negative *Staphylococcus***	12·2%	10·1%	9·6% (6·7%, 12·9%)	<0·0001**	89·9%	0·26 (0·11,0·41)	0·80
Corynebacterium	9·4%	8·6%	8·1% (5·4%, 11·2%)	<0·0001**	91·4%	0·13 (-0·01,0·28)	0·83
Pseudomonas aeruginiosa	8·6%	4·1%	0·0% (-2·0%, 2·0%)	1·0000	95·9%	0·67 (0·52,0·82)	0·92
***Staphylococcus aureus* (exc· MRSA)**	35·7%	8·1%	0·0% (-2·8%, 2·8%)	1·0000	91·9%	0·81 (0·75,0·87)	0·84

*Tissue – swab, **exact p-value / CI

For peer review only

Table 4 Multinomial and ordinal regression models for individually fitted baseline factors

		Odds Ratio (95% CI)	AIC	Reduction in -2LogL	DF	P-value
**Multinomial Summary of Isolates**	Both swab and tissue report the same pathogens vs:					
**Null Model**			941·29			
**Ulcer Type**[1]			945·72	1·570	3	0.666
Any Ischemia vs Neuropathic only	Swab > pathogens compared to the tissue	1·03(0·48,2·20)				
Any Ischemia vs Neuropathic only	Tissue > pathogens compared to the swab	0·86(0·53,1·40)				
Any Ischemia vs Neuropathic only	Swab and tissue report totally different pathogens	0·68(0·35,1·31)				
**Ulcer Grade**			949·16	4·125	6	0·660
Grade 2 vs Grade 1	Swab > pathogens compared to the tissue	0·68(0·26,1·78)				
Grade 2 vs Grade 1	Tissue > pathogens compared to the swab	1·08(0·60,1·93)				
Grade 2 vs Grade 1	Swab and tissue report totally different pathogens	1·14(0·51,2·54)				
Grade 3/4/5 v Grade 1	Swab> pathogens compared to the tissue	1·28(0·52,3·11)				
Grade 3/4/5 v Grade 1	Tissue > pathogens compared to the swab	1·60(0·87,2·95)				
Grade 3/4/5 v Grade 1	Swab and tissue report totally different pathogens	1·55(0·69,3·45)				
**Previous antibiotic therapy**[1]			946·28	1·005	3	0·800
Yes vs No	Swab > pathogens compared to the tissue	0·80(0·36,1·80)				
Yes vs No	Tissue > pathogens compared to the swab	1·14(0·69,1·89)				
Yes vs No	Swab and tissue report totally different pathogens	1·10(0·56,2·16)				
**Antimicrobial Dressing**[1]			943·44	3·850	3	0.278
Yes vs No	Swab > pathogens compared to the tissue	1·13(0·51,2·51)				
Yes vs No	Tissue > pathogens compared to the swab	0·69(0·40,1·19)				
Yes vs No	Swab and tissue report totally different pathogens	1·38(0·66,2·89)				

For peer review only - http://bmjopen.bmj.com/site/about/guidelines.xhtml

**Wound Duration (Median split)** [1]			941·48	5·802	3	0·121
<56 days vs >=56 days	Swab > pathogens compared to the tissue	0·94 (0·43, 2·04)				
<56 days vs >=56 days	Tissue > pathogens compared to the swab	1·75 (1·08, 2·86)*				
<56 days vs >=56 days	Swab and tissue report totally different pathogens	1·14 (0·59, 2·17)				
**Log Wound Duration (Continuous)** [1]			944·97	2·318	3	0.509
	Swab > pathogens compared to the tissue	0·95(0·72,1·25)				
	Tissue > pathogens compared to the swab	0·88(0·74,1·04)				
	Swab and tissue report totally different pathogens	0·93(0·74,1·18)				
**Ordinal Summary of Isolates**						
**Null Model**			917·72			
**Ulcer Type**[1]**:**Any Ischaemia vs Neuropathic only		0·90(0·61,1·33)	919·45	0·271	1	0·603
**Ulcer Grade**			920·16	1·559	2	0.459
Grade 2 vs Grade 1		1·33(0·82,2·15)				
Grade 3, 4, or 5 vs Grade 1		1·27(0·78,2·07)				
**Previous antibiotic therapy**[1]**:**Yes vs No		1·25(0·81,1·91)	918·56	1·154	1	0·283
**Antimicrobial Dressing**[1]**:** Yes vs No		0·76(0·49,1·18)	918·16	1·553	1	0·213
**Wound Duration (Median split)**[1]**:** <56 days vs >=56 days		1·56(1·05,2·33)	914·62	5·097	1	0·024**
**Log Wound Duration (Continuous)**[1]		0·92(0·80,1·05)	918·15	1·571	1	0·210

Note: Based on the evaluable population N=395 / [a]Smaller is better / [1] factors with missing data from the 28 (7·1%) patients with at least one missing data item / **significant at the 5% level

Figure legend: Figure 1 Study Recruitment Diagram

For peer review only - http://bmjopen.bmj.com/site/about/guidelines.xhtml

Figure 1: Study Recruitment Diagram

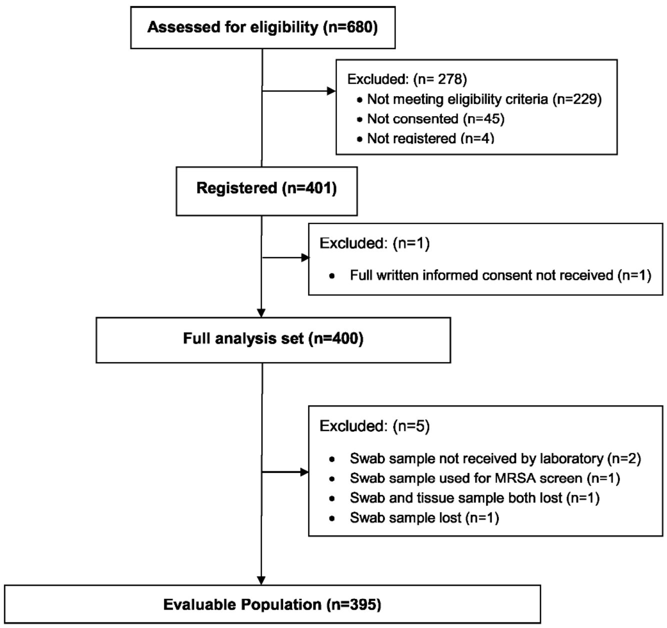

Figure 1 Study Recruitment Diagram

209x297mm (300 x 300 DPI)

STROBE 2007 (v4) Statement—Checklist of items that should be included in reports of *cross-sectional studies*

Section/Topic	Item #	Recommendation	Reported on page #
**Title and abstract**	1	(*a*) Indicate the study's design with a commonly used term in the title or the abstract	1
		(*b*) Provide in the abstract an informative and balanced summary of what was done and what was found	2
**Introduction**			
Background/rationale	2	Explain the scientific background and rationale for the investigation being reported	4
Objectives	3	State specific objectives, including any prespecified hypotheses	5
**Methods**			
Study design	4	Present key elements of study design early in the paper	5
Setting	5	Describe the setting, locations, and relevant dates, including periods of recruitment, exposure, follow-up, and data collection	5; 9
Participants	6	(*a*) Give the eligibility criteria, and the sources and methods of selection of participants	5
Variables	7	Clearly define all outcomes, exposures, predictors, potential confounders, and effect modifiers. Give diagnostic criteria, if applicable	5, 6
Data sources/ measurement	8*	For each variable of interest, give sources of data and details of methods of assessment (measurement). Describe comparability of assessment methods if there is more than one group	5-7
Bias	9	Describe any efforts to address potential sources of bias	*5-6*
Study size	10	Explain how the study size was arrived at	7
Quantitative variables	11	Explain how quantitative variables were handled in the analyses. If applicable, describe which groupings were chosen and why	7-8
Statistical methods	12	(*a*) Describe all statistical methods, including those used to control for confounding	8-9
		(*b*) Describe any methods used to examine subgroups and interactions	8
		(*c*) Explain how missing data were addressed	8
		(*d*) If applicable, describe analytical methods taking account of sampling strategy	8
		(*e*) Describe any sensitivity analyses	N/A
**Results**			

For peer review only

Participants	13*	(a) Report numbers of individuals at each stage of study—eg numbers potentially eligible, examined for eligibility, confirmed eligible, included in the study, completing follow-up, and analysed	8
		(b) Give reasons for non-participation at each stage	8; figure 1
		(c) Consider use of a flow diagram	Figure 1
Descriptive data	14*	(a) Give characteristics of study participants (eg demographic, clinical, social) and information on exposures and potential confounders	9
		(b) Indicate number of participants with missing data for each variable of interest	22 (Table 1)
Outcome data	15*	Report numbers of outcome events or summary measures	10-12
Main results	16	(*a*) Give unadjusted estimates and, if applicable, confounder-adjusted estimates and their precision (eg, 95% confidence interval). Make clear which confounders were adjusted for and why they were included	10-11; 26 (Table 4)
		(*b*) Report category boundaries when continuous variables were categorized	11; 26
		(*c*) If relevant, consider translating estimates of relative risk into absolute risk for a meaningful time period	N/A
Other analyses	17	Report other analyses done—eg analyses of subgroups and interactions, and sensitivity analyses	11-12
**Discussion**			
Key results	18	Summarise key results with reference to study objectives	12-13
Limitations	19	Discuss limitations of the study, taking into account sources of potential bias or imprecision. Discuss both direction and magnitude of any potential bias	14
Interpretation	20	Give a cautious overall interpretation of results considering objectives, limitations, multiplicity of analyses, results from similar studies, and other relevant evidence	13-15
Generalisability	21	Discuss the generalisability (external validity) of the study results	14
**Other information**			
Funding	22	Give the source of funding and the role of the funders for the present study and, if applicable, for the original study on which the present article is based	16

*Give information separately for cases and controls in case-control studies and, if applicable, for exposed and unexposed groups in cohort and cross-sectional studies.

Note: An Explanation and Elaboration article discusses each checklist item and gives methodological background and published examples of transparent reporting. The STROBE checklist is best used in conjunction with this article (freely available on the Web sites of PLoS Medicine at http://www.plosmedicine.org/, Annals of Internal Medicine at http://www.annals.org/, and Epidemiology at http://www.epidem.com/). Information on the STROBE Initiative is available at www.strobe-statement.org.

For peer review only
